# “MrgprA3 neurons selectively control myeloid-derived cytokines for IL-17 dependent cutaneous immunity”

**DOI:** 10.21203/rs.3.rs-3644984/v1

**Published:** 2023-11-30

**Authors:** Juan M. Inclan-Rico, Camila M. Napuri, Cailu Lin, Li-Yin Hung, Annabel A. Ferguson, Qinxue Wu, Christopher F. Pastore, Adriana Stephenson, Ulrich M. Femoe, Heather L. Rossi, Danielle R. Reed, Wenqin Luo, Ishmail Abdus-Saboor, De’Broski R. Herbert

**Affiliations:** 1 Department of Pathobiology, School of Veterinary Medicine, University of Pennsylvania, Philadelphia, Pennsylvania, USA.; 2 Monell Chemical Senses Center, Philadelphia, Pennsylvania, USA.; 3 Department of Neuroscience, School of Medicine, University of Pennsylvania, Philadelphia, Pennsylvania, USA.; 4 Institute for Regenerative Medicine, School of Medicine, University of Pennsylvania, Philadelphia, Pennsylvania, USA.; 5 Department of Biological Sciences, Zuckerman Mind, Brain, Behavior Institute, Columbia University, New York, New York, USA.

**Keywords:** MrgprA3, macrophages, helminths, IL-33, type 17 responses, keratinization

## Abstract

Skin employs interdependent cellular networks to facilitate barrier integrity and host immunity through ill-defined mechanisms. This study demonstrates that manipulation of itch-sensing neurons bearing the Mas-related G protein-coupled receptor A3 (MrgprA3) drives IL-17+ γδ T cell expansion, epidermal thickening, and resistance to the human pathogen *Schistosoma mansoni* through mechanisms that require myeloid antigen presenting cells (APC). Activated MrgprA3 neurons instruct myeloid APCs to downregulate interleukin 33 (IL-33) and up-regulate TNFα partially through the neuropeptide calcitonin gene related peptide (CGRP). Strikingly, cell-intrinsic deletion of IL-33 in myeloid APC basally alters chromatin accessibility at inflammatory cytokine loci and promotes IL-17/23-dependent epidermal thickening, keratinocyte hyperplasia, and resistance to helminth infection. Our findings reveal a previously undescribed mechanism of intercellular cross-talk wherein “itch” neuron activation reshapes myeloid cytokine expression patterns to alter skin composition for cutaneous immunity against invasive pathogens.

## One sentence summary

MrgprA3 neurons regulate myeloid cells to promote cutaneous IL-17-dependent host protective responses.

## Introduction

Skin architecture contains a complex array of hematopoietic (e.g., myeloid, lymphoid) and non-hematopoietic (e.g., keratinocyte, sensory neuron) cell types that control homeostasis and immunity. Skin-resident myeloid antigen presenting cell (APC) subsets including tissue macrophages, type 1 conventional dendritic cells (cDC1), cDC2, and Langerhan cells (LCs) can initiate cutaneous inflammation in response to mechanical, chemical, or biological stimuli^1^. However, under basal conditions, such responses are held in check to allow for homeostatic interactions with the microbiota through processes like keratinization^2^. Through a constitutive process, basal keratinocyte turnover drives cellular transition through well-defined epidermal layers into the outermost water-impermeable barrier, the cornified envelope. Epidermal integrity relies on the adequate cross-linking of several extracellular matrix proteins that make up the layers of the cornified envelope including loricrin, involucrin, and desmosomal components, such as desmoglein-1^2^. Myeloid cells can facilitate keratinocyte turnover and effector functions through secreting pro-inflammatory cytokines (e.g., Interleukins IL-1β, IL-6, IL-23, and Tumor Necrosis Factor α, TNFα) that elicit IL-17-mediated γδ T cell responses that drive cutaneous inflammation and/or wound healing^3, 4, 5^. But how the skin composition rapidly transitions between basal regulatory processes vs. host-protective mechanisms directed against pathogens remain unknown.

Whether immunity against skin-penetrating parasitic helminths like trematodes (blood flukes) or nematodes (hookworms) can be initiated by interactions between myeloid cells and other tissue resident populations like sensory neurons is unexplored. Most sensory afferents pertinent to cutaneous infections can be broadly grouped into a class of nociceptive neurons that sense pain and itch through expression of the non-selective cation channel transient receptor potential vanilloid member 1 (TRPV1)^6^. Neurons that sense itch can also be defined by expression of members of the Mas-related G protein-coupled receptor family (Mrgpr) and are found in the dorsal root and trigeminal ganglia^7^. In rodents, these include MrgprD^8, 9, 10^, which mediates beta-alanine-evoked itch, and MrgprA3, which mediates chloroquine(CQ)-evoked itch^11^. This family of sensory neurons is largely understudied with respect to their communication with other cell types in the skin as compared to TRPV1. TRPV1+ neurons can evoke IL-17-dependent cutaneous inflammation via Calcitonin gene related peptide (CGRP) release that has been shown to stimulate pro-inflammatory cytokine release by DCs^12, 13, 14, 15^. Further, TRPV1+ neurons promote allergic inflammation by their release of Substance P (SP); which induces dendritic cell migration via the MrgprA1 receptor and mast cell degranulation by acting on MrgprB2^16, 17^. TRPV1+ neurons are largely distinct from MrgprD+ neurons but have partial overlap of expression with MrgprA3+ neurons^8, 10, 11, 18^. Nonetheless, single cell RNAseq studies recently defined MrgprA3+ neurons as a unique sensory neuronal subset proposed to have unique functions^19, 20, 21, 22^. For example, MrgprA3+ neurons can secrete CGRP, but they do not produce SP^11, 23^, whereas MrgprD+ neurons do not express either of these canonical neuropeptides^24^. While (MrgprD) has been shown to restrain mast cell degranulation during homeostasis^25^, the biological roles served by skin MrgprA3 neurons under basal or infectious conditions remain poorly understood.

Cutaneous inflammation caused by allergens or pathogens can initiate from tissue injury, through release of damage associated molecular patterns (DAMPs) including adenosine triphosphate (ATP) or High mobility group box 1 protein (HMGB1) and alarmin cytokines like IL-33. IL-33 is an IL-1 family cytokine constitutively held in the nucleus of epithelial, endothelial, and stromal cell types (i.e., fibroblasts) that can be released upon cell death, which allows it to engage its receptor T1/ST2 expressed primarily on ILC2s, type 2 helper (T_H_2) cells, Tregs, mast cells, basophils, dendritic cells, and macrophages^26^. Importantly, it has become increasingly clear that hematopoietic cells including mast cells and cDC can also serve as biologically important sources of IL-33 that promote anaphylaxis and GATA3+Treg maintenance^27, 28, 29^. IL-33 release in skin can drive dry skin itch through a sensory neuron ST2-dependent mechanism^30^. However, it remains obscure whether myeloid cell-intrinsic IL-33 regulates inflammation and whether neuronal activity can feedback on myeloid cells to regulate IL-33 production has not been considered.

This study reveals that itch-sensing MrpgrA3 neurons innervate the skin near areas where myeloid APC reside and that optogenetic or pharmacological stimulation of these rare neurons selectively downregulates cell-intrinsic IL-33 expression in tissue macrophages and cDC2, but not in skin fibroblasts. This ability of MrgprA3 neurons to mediate IL-33 suppression requires CGRP, resulting in IL-17-inducing cytokine release (IL-1β and TNFα), skin thickening, and innate resistance to skin infection with the helminth *Schistosoma mansoni*. Accordingly, MrgprA3 neuron ablation augments IL-33 expression in dermal APCs, impairs skin IL-17 responses, and hampers cutaneous anti-schistosome immunity. Strikingly, genetic deficiency of IL-33 only in the myeloid compartment phenocopies MrgprA3 activation in the skin, with excessive keratinization, epidermal thickening, and innate resistance to *S. mansoni*, all of which relied upon IL-17 dominated inflammation. Critically, MrgprA3 neuron stimulation in mice lacking myeloid-IL-33 fails to enhance skin inflammation or anti-helminth resistance, implying that MrgprA3 neurogenic inflammation is mechanistically linked to suppression of myeloid-intrinsic IL-33. Indeed, employ of scRNAseq, ATAC-seq, and *in vitro* approaches reveal that myeloid-intrinsic IL-33 expression governs the transcriptional and chromatin landscape of macrophages and cDC, the lack of which lowers the threshold of pro-inflammatory cytokine gene expression and release. Collectively, this work suggests a neuroimmune circuit wherein a small class of itch-specific sensory neurons can selectively re-organize myeloid cytokine gene expression patterns to rapidly drive IL-17-mediated cutaneous immunity.

## Results

### MrgprA3 neurons control cutaneous anti-helminth immunity

**Extended Data Figure 1.**
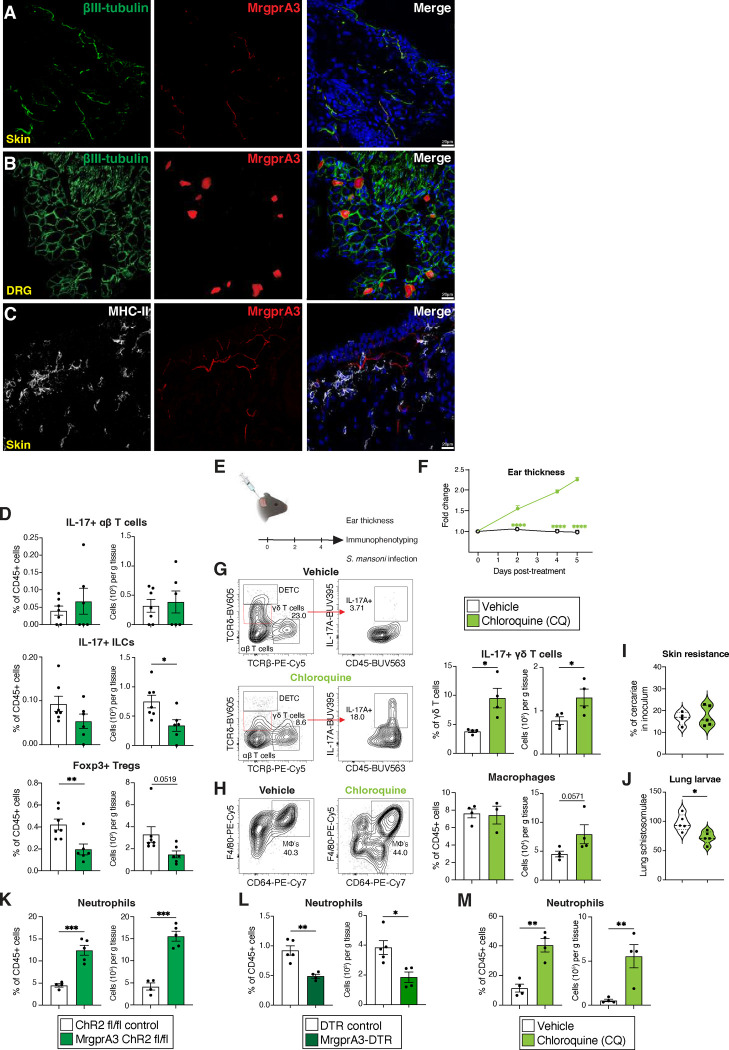
MrgprA3 neurons control skin immunity against S. mansoni. (**A,B**), Individual channels of IFA staining of MrgprA3 and βIII tubulin in unmanipulated dorsal skin and dorsal root ganglia (DRG) sections of MrgprA3^Cre+/−^:Rosa26^tdTomato^ mice. (**C**) Individual channels of MHC-II, and MrgprA3 in unmanipulated abdominal skin sections of MrgprA3^Cre+/−^:Rosa26^tdTomato^ mice. (**D**), Proportions and absolute cell numbers of skin IL-17+ αβ T cells, IL-17+ ILCs, and Foxp3+ Tregs were quantified by flow cytometry of control or MrgprA3-ChR2 mice 5 days post-optogenetic stimulation. (**E**), Experimental approach for intradermal injection with MrgprA3 agonist, chloroquine (CQ) followed by measurement of ear thickness, immunophenotype, and resistance against *S. mansoni* infection. (**F**), Ear thickness was measured daily during CQ treatment assays. (**G,H**), Representative counterplots and quantification of skin IL-17+ γδ T cells and macrophages by flow cytometry of wildtype mice treated with vehicle or CQ. (**I,J**), Percentage of non-penetrating cercariae and D6 lung larval burden from vehicle or CQ-treated mice prior to *S. mansoni* skin exposure. (**K-M**), One day after *S. mansoni* challenge, neutrophil ear skin responses were evaluated in ChR2 control or MrgprA3-ChR2 mice; vehicle or CQ-treated wildtype mice; and control or MrgprA3-DTR mice. *P* values were determined by two-tailed Student’s t-tests or Two-way ANOVA with post hoc correction. *P<0.05, **P<0.01, ***P<0.001. Representative of 2–3 independent experiments, each with ≥4 biological replicates.

**Figure 1.**
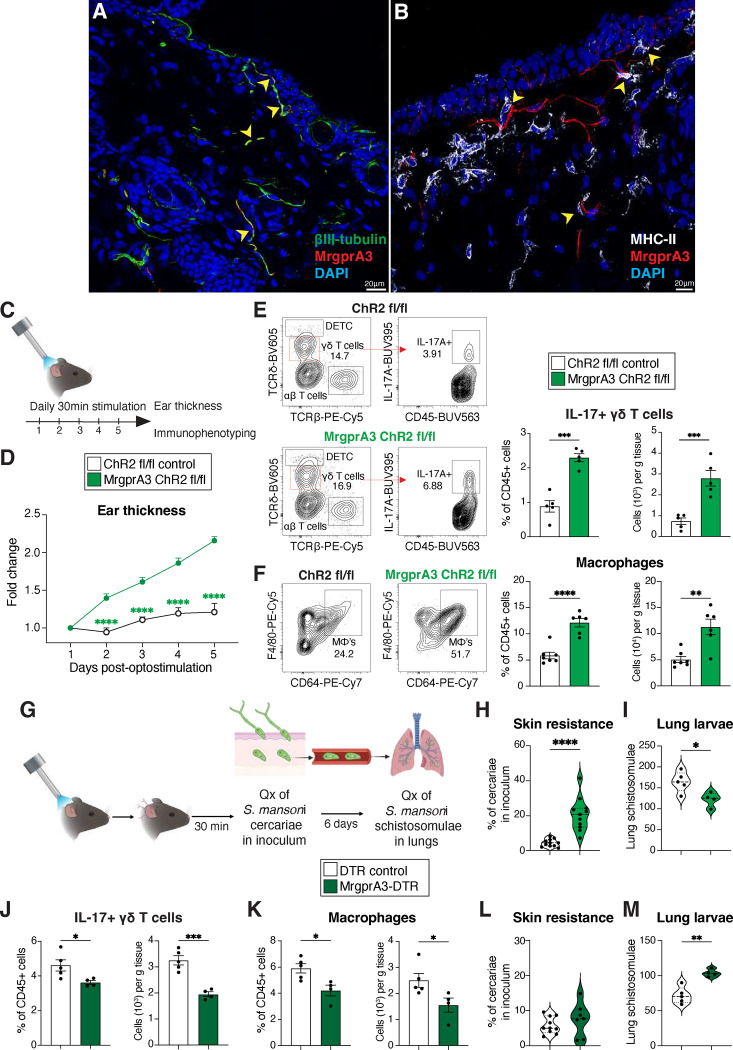
MrgprA3 neurons are essential and sufficient for cutaneous immunity against S. mansoni. (**A**), IFA staining of MrgprA3 and βIII tubulin in unmanipulated dorsal skin sections of MrgprA3^Cre+/−^:Rosa26^tdTomato^ mice. Yellow arrows indicate MrgprA3+ neurons. (**B**) IFA staining of MHC-II and MrgprA3 in naïve abdominal skin sections of MrgprA3^Cre+/−^:Rosa26^tdTomato^ mice. Yellow arrows indicate MHC-II+ cells closely located to MrgprA3+ fibers. (**C**), Experimental approach for optogenetic ear stimulation of MrgprA3-ChR2 followed by evaluation of ear thickness and immunophenotype. (**D**), Ear thickness was measured daily during optogenetic stimulation. (**E,F**), Skin IL-17+ γδ T cells and macrophages were quantified by flow cytometry of control or MrgprA3-ChR2 mice 5 days post-optogenetic stimulation. (**G**), Life cycle and experimental approach to evaluate lung parasite load at after skin exposure with 150–200 *Schistosoma mansoni* cercariae. (**H**), Percentage of non-penetrating cercariae from ChR2 control or MrgprA3-ChR2 mice that were exposed to 30-min blue light stimulation prior to ear cercarial exposure. (**I**), Lung schistosomulae of light stimulated ChR2 control, or MrgprA3-ChR2 mice 6 days post-infection. (**J,K**), Skin IL-17+ γδ T cells and macrophages were quantified by flow cytometry of control or MrgprA3-DTR mice after 3 weeks of DT systemic administration. (**L**), Percentage of non-penetrating cercariae from DTR control or MrgprA3-DTR mice that were exposed to 150–200 *S. mansoni* cercariae. (**M**), Lung schistosomulae of light stimulated DTR control, or MrgprA3-DTR mice 6 days post-infection. *P* values were determined by two-tailed Student’s t-tests, One-way ANOVA or Two-way ANOVA with post hoc correction. *P<0.05, **P<0.01, ***P<0.001, ****P<0.0001. Representative of 2–3 independent experiments, each with ≥4 biological replicates.

Nociceptors, i.e. sensory neurons that promote pain and itch in response to noxious stimuli, have recently emerged as central regulators of cutaneous immunity in the context of fungal and bacterial infection as well as psoriasis^12, 13, 14, 15^. Specifically, nociceptors were shown to alter dendritic cell responses via their secretion of the neuropeptide CGRP^15, 31^. However, whether itch-inducing MrgprA3 neurons can alter the cutaneous immune landscape and affect the outcome of infection with skin-penetrating helminths is unknown. To explore the feasibility of this idea, MrgprA3-tDtomato reporter mice were used to assess the proximity of MHC-II+ antigen presenting cells (APCs) to itch-transmitting MrgprA3 neurons in the skin^11, 32^. As expected, MrgprA3+ nerves were sparse and colocalized with the pan-neuronal marker βIII-tubulin+ in both skin and dorsal root ganglia (Fig. 1a,b and Extended Data Fig.1a,b) and were associated with MHCII+ cells in naïve skin (Fig. 1c and Extended Data Fig.1c). To address the possibility that itch-inducing MrgprA3 neurons could influence the skin immune landscape, an optogenetic strategy was used to selectively activate MrgprA3 neurons engineered to express channelrhodopsin 2 (ChR2) using 473 nm blue light as previously shown^33, 34^. Using previously established protocols ^12^, ear skin of control MrgprA3^Cre^ neg ChR2 (ChR2) or MrgprA3^Cre^ pos ChR2 (MrgprA3-ChR2) mice was exposed to blue light for 30 mins/day for 5 consecutive days (Fig. 1c). This strategy resulted in ear thickening and increased frequency and number of IL-17+ γδ T cells and skin macrophages, (Fig. 1d–f). Conversely, other populations of IL-17 expressing lymphocytes cell such as ILCs and αβ T cells were not significantly changed, while Foxp3+ T regulatory cells were moderately diminished when MrgprA3 neurons were stimulated (Extended Data Fig.1d). To account for potential off-target effects of transgenic expression of ChR2, a complementary approach was taken by administering intradermal chloroquine (CQ), an itch-inducing MrgprA3 agonist^11^, in the ear every second day (Extended Data Fig.1e). Consistent with the results of optogenetic stimulation, CQ administration also increased ear thickening, expansion of IL-17+ γδ T cells, and a trend in increased numbers of skin macrophages at the injection site (Extended Data Fig.1f–h).!

There is surprisingly little known about whether cutaneous myeloid subsets contribute to mechanisms that repel skin invasion by large metazoan parasites^35^. The human pathogen *S. mansoni* is a trematode blood fluke that rapidly penetrates the skin, travels through the blood stream and enters the lungs by day 6 post-infection in mice^36^ (Fig. 1g). Given that skin architecture and cellular composition was reshaped by stimulation of MrgprA3 neurons, we postulated that such neurogenic inflammation could potentially enhance innate resistance to *S. mansoni* in an otherwise naïve host, potentially affecting subsequent larval migration into the lung. Indeed, mice subjected to MrgprA3 neuron optogenetic activation had significantly augmented resistance to skin penetration by *S. mansoni* with significantly fewer larvae recovered from the lung (Fig. 1h,i). Optogenetic A3 neuron activation also increased the proportion and number of neutrophils recruited to the skin 1 day post-*S. mansoni* infection (Extended Data Fig.1k). Although CQ administration did not significantly block skin entry of *S. mansoni* cercariae, CQ pre-treatment increased skin neutrophils 1 day post-infection and significantly reduced the numbers of larvae that reached the lung by day 6 (Extended Data Fig.1i,j,m). To complement these gain-of-function studies, a loss of function approach was employed by interbreeding MrgprA3Cre and loxPSTOPloxP DTR mouse strains to generate animals allowing selectively depletion of MrgprA3 neurons (MrgprA3Cre-DTR) after 3 weeks of DT administration as previously described^11^. Critically, MrgprA3Cre-DTR mice treated with DT had significantly reduced numbers and proportions of IL-17+ γδ T cells and macrophages in the skin (Fig. 1j,k). Depletion of MrgprA3 neurons also did not significantly impact skin penetration of *S. mansoni* larvae, but markedly reduced the proportion and number of skin neutrophils 1 day post-infection and significantly increased the number of larvae that entered lung tissue by day 6 (Fig. 1l,m; Extended Data Fig.1l). Collectively, this indicated that itch-sensing MrgprA3 neurons were both capable and required to drive cutaneous anti-helminth immunity, potentially through local activation of macrophages, neutrophils, and IL-17+ γδ T cells.

### MrgprA3 neurons promote macrophage-mediated inflammation to promote resistance against helminth skin entry.

**Extended Data Figure 2.**
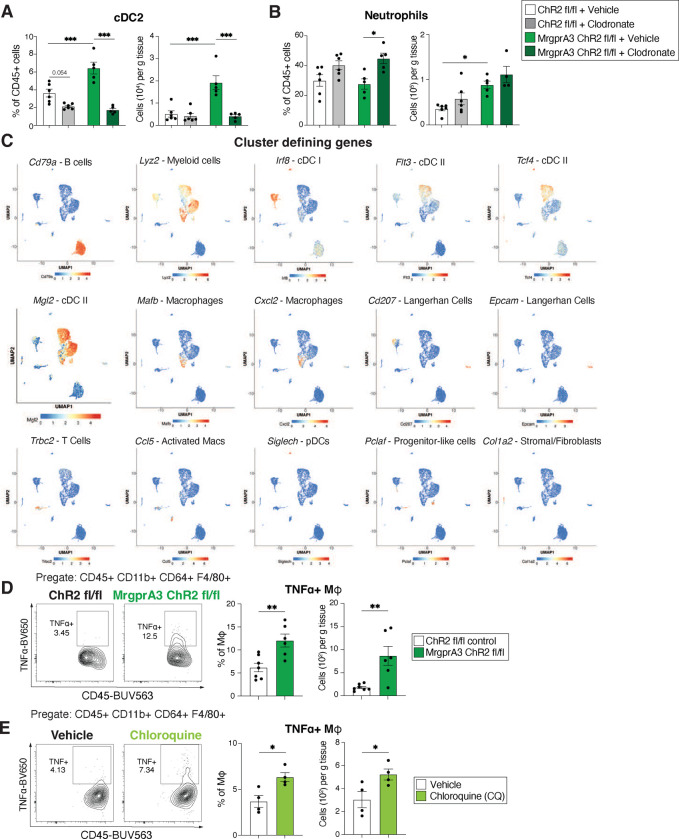
MrgprA3 neurons control skin macrophage responses. (**A,B**), Evaluation of skin cDC2 and neutrophil cell populations in vehicle- or clodronate treated control or MrgprA3-ChR2 mice following optogenetic stimulation. (**C**), UMAP plots illustrating expression of signature genes in defined clusters of cells generated by single cell RNA-sequencing of skin-resident live CD11c+ MHC-II+ populations sort-purified from naïve mice. (**D,E**), Representative counterplots and quantification of skin TNFα+ macrophages following optogenetic or pharmacological activation of MrgprA3 neurons. *P* values were determined by two-tailed Student’s t-tests or One-way ANOVA with post hoc correction. *P<0.05, **P<0.01, ***P<0.001. Representative of 2–3 independent experiments, each with ≥4 biological replicates.

**Figure 2.**
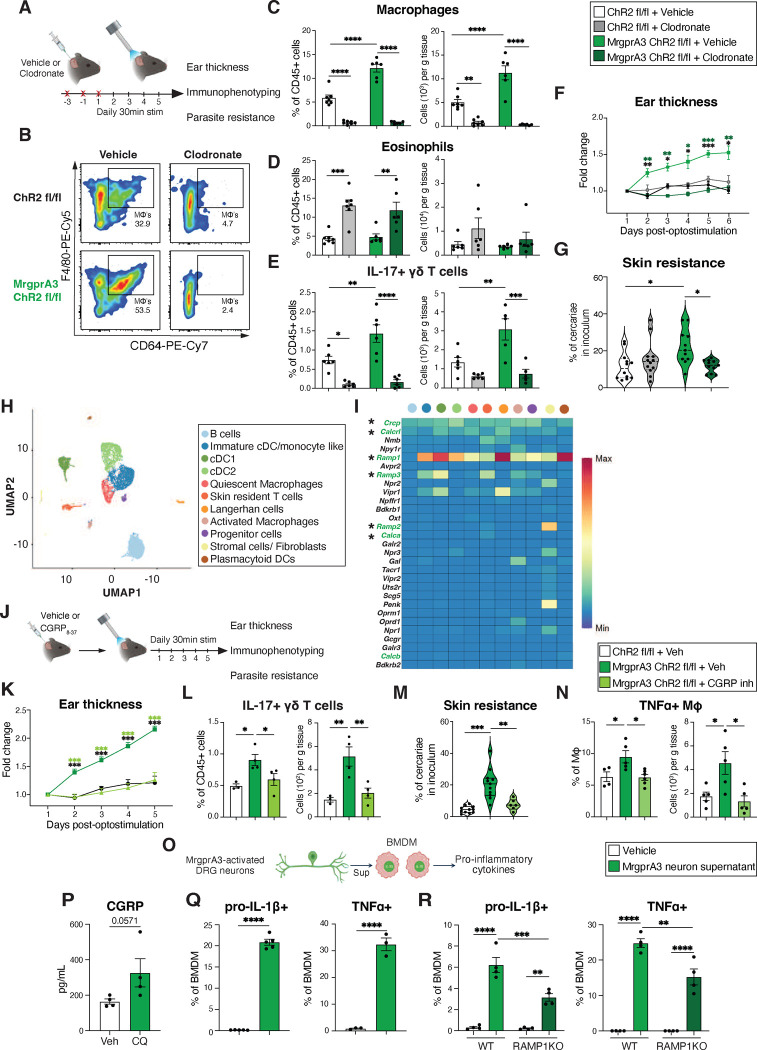
MrgprA3+ neurons promote skin immunity against S. mansoni through macrophages and CGRP. (**A**), Experimental approach for vehicle- or clodronate-loaded liposomes intradermal injection in the right or left ear of control or MrgprA3-ChR2 mice prior to optogenetic ear stimulation. (**B-E**), Evaluation of skin macrophage, eosinophil, and IL-17+ γδ T cell populations in vehicle- or clodronate treated control or MrgprA3-ChR2 mice. (**F**), Ear thickness was measured daily during optogenetic stimulation. (**G**), Percentage of non-penetrating cercariae from ChR2 control or MrgprA3-ChR2 mice treated with vehicle- or clodronate-loaded liposomes that were exposed to 30-min blue light stimulation prior to ear *S. mansoni* exposure. (**H**), Uniform Manifold Approximation and Projection (UMAP) plot illustrating defined clusters of cells generated by single cell RNA-sequencing of skin-resident live CD11c+ MHC-II+ populations sort-purified from naïve mice. (**I**), Heatmap showing expression of neuropeptide ligands and receptors in cell clusters. (**J**), Experimental approach for CGRP inhibition by intradermal administration of CGRP antagonist, CGRP_8–37_, prior to optogenetic ear stimulation of MrgprA3-ChR2 mice. (**K**), Ear thickness of control, vehicle-treated MrgprA3-ChR2, or MrgprA3-ChR2 treated with CGRP inhibitor prior to post-optogenetic stimulation for 5 days. (**L**), IL-17+ γδ T cells and (**N**), TNFα+ macrophages from control, vehicle-treated MrgprA3-ChR2, and CGRP inhibitor-treated MrgprA3-ChR2 mice were quantified by flow cytometry 5 days post-optogenetic stimulation. (**M**), Percentage of non-penetrating cercariae from control or MrgprA3-ChR2 mice were treated with vehicle or CGRP inhibitor prior to post-optogenetic stimulation followed by parasite exposure. (**O**), Bone marrow-derived macrophages (BMDMs) were exposed to cell-free supernatants derived from dorsal root ganglia (DRG) neurons stimulated with MrgprA3 ligand chloroquine (CQ) for 1 hour. Expression of pro-inflammatory cytokines was evaluated 24hrs post-exposure. (**P**), Calcitonin gene related peptide (CGRP) levels in neuron supernatant were evaluated after 1h of CQ treatment. (**Q**), Pro-IL-1β+ and TNFα+expression of control or BMDMs exposed to supernatants derived from CQ-stimulated DRG neurons. (**R**), BMDMs from control or RAMP1KO mice were exposed to CQ-activated neuron supernatant for 24hrs and the expression of pro-IL-1β+ and TNFα+was evaluated. *P* values were determined by two-tailed Student’s t-tests, One-way ANOVA or Two-way ANOVA with post hoc correction. *P<0.05, **P<0.01, ***P<0.001, ****P<0.0001. Representative of 2–3 independent experiments, each with ≥3 biological replicates.

Professional phagocytes such as dermal dendritic cells and macrophages can promote IL-17+ γδ T cell responses and recruitment of neutrophils into the skin^37, 38^. In mucosal tissues such as the lung and intestine, eosinophils and neutrophils were proposed to trap and kill helminth larvae^39, 40^. Thus, we tested if skin phagocytes were required for activated MrgprA3 neurons to alter skin architecture and drive anti-helminth immunity. Control or MrgprA3-ChR2 mice were injected intradermally in one ear with vehicle liposomes and in the other ear with clodronate-loaded liposomes at d-3 and d-1 prior to light stimulation followed by evaluation of ear thickness, dermal immune cell composition, and susceptibility to skin penetration via *S. mansoni* (Fig. 2a). Clodronate injection ablated skin macrophages (Fig. 2b,c) and cDC2s (Extended Data Fig.2a) in both control and MrgprA3-ChR2 mice whereas neutrophil and eosinophil populations were increased to some extent by clodronate treatment (Fig. 2d; Extended Data Fig.2b). Importantly, IL-17+ γδ T cells, ear swelling, and resistance to skin penetration by *S. mansoni* larvae induced by optogenetic activation of MrgprA3 neurons were all reversed by clodronate administration (Fig. 2e–g). These data indicated that macrophages and dendritic cells functioned downstream of MrgprA3 neurons.

To investigate whether MrgprA3 neurons could communicate with skin myeloid subsets through neuropeptides, scRNAseq analysis was performed on sort-purified CD45+ CD11c+ MHCII+ cells from naïve abdominal skin. Eleven clusters were defined based on their expression of lineage-specific genes including B cells, immature/monocyte like cDC, cDC1, cDC2, quiescent macrophages, skin resident T cells, Langerhan cells, activated macrophages, progenitor cells, stromal cells/fibroblasts, and pDCs (Fig. 2h; Extended Data Fig.2c). Virtually, all skin-resident APCs expressed diverse neuropeptide receptors at baseline (Fig. 2i), most notably the subunits of the CGRP receptor: *Crcp* (Calcitonin gene-related peptide-receptor component protein), *Calcrl* (Calcitonin Receptor-like Receptor), and *Ramp1* (Receptor activity modifying protein 1), and a related subunit *Ramp3*, which forms the adrenomedullin 2/intermedin receptor when paired with *Calcrl^41^*. Myeloid cell clusters did not exhibit high expression of *Calca* or *Calcb*, which encode CGRPα and CGRPβ respectively, making autoregulation through intrinsic expression of ligands for these receptors unlikely. Furthermore, *Tacr1* (the SP or tachykinin 1 receptor) was not enriched in any of these populations (Fig. 2i). Immature/monocyte like DC, cDC2, and Langerhan cell clusters were enriched for *Vipr1* (vasoactive intestinal peptide receptor 1) whereas stromal cells/fibroblasts were enriched for *Ramp2* (a component of adrenomedullin 1 or amylin 2 receptors*)*, *Npr1*(Natriuretic peptide receptor A or 1/Guanylate cyclase A), and *Npr3* (Natriuretic peptide receptor C or 3/Guanylate cyclase C) (Fig. 2i). Of note, we did not find *Tacr2, Mrgpra1,* or *Mrgprb2* cell cluster expression our scRNAseq analysis (data not shown) despite their essential roles in mast cell and neutrophil responses^16, 25, 42, 43^. Based on these data, we postulated that activated skin MrgprA3 neurons, known to express high levels of *Calca*, potentially communicated with neighboring myeloid APC through CGRP release.

This CGRP hypothesis was tested by treatment of mice undergoing MrgprA3+ neuron activation with the CGRP antagonist, CGRP_8–37_ (Fig. 2j). As predicted, CGRP inhibition prevented ear swelling, blunted IL-17+ γδ T cell accumulation, and impaired resistance to skin penetration in the context of MrgprA3 neuron activation (Fig. 2k–m). Accordingly, the increase in frequency and number of TNFα+ macrophages elicited by optogenetic or pharmacological stimulation of MrgprA3 neurons was impaired by CGRP inhibitor treatment (Fig. 2n, Extended Data Fig.2d,e). To ask if soluble mediator release from MrgprA3 neurons directly stimulated macrophage responses, dorsal root ganglia (DRG) neurons were isolated, cultured, and activated with CQ for 1hr, supernatants collected and used to stimulate bone marrow-derived macrophages (BMDMs) for 24hrs followed by cytokine analysis (Fig. 2o). As expected, CQ treatment moderately increased CGRP levels in the neuronal supernatant (Fig. 2p). Exposure of BMDMs to supernatants from CQ-treated neurons significantly increased expression of pro-IL-1β and TNFα (Fig. 2q). Similar treatment of BMDMs lacking the CGRP receptor subunit RAMP1 with CQ-induced neuron supernatant induced significantly less pro-IL-1β and TNFα compared to WT BMDMs (Fig. 2r). Taken together, these results suggested that CGRP was partially responsible for the effects of MrgprA3 neurons on macrophage cytokine expression.

### MrgprA3 neurons selectively downregulate myeloid cell expression of IL-33

**Extended Data Figure 3.**
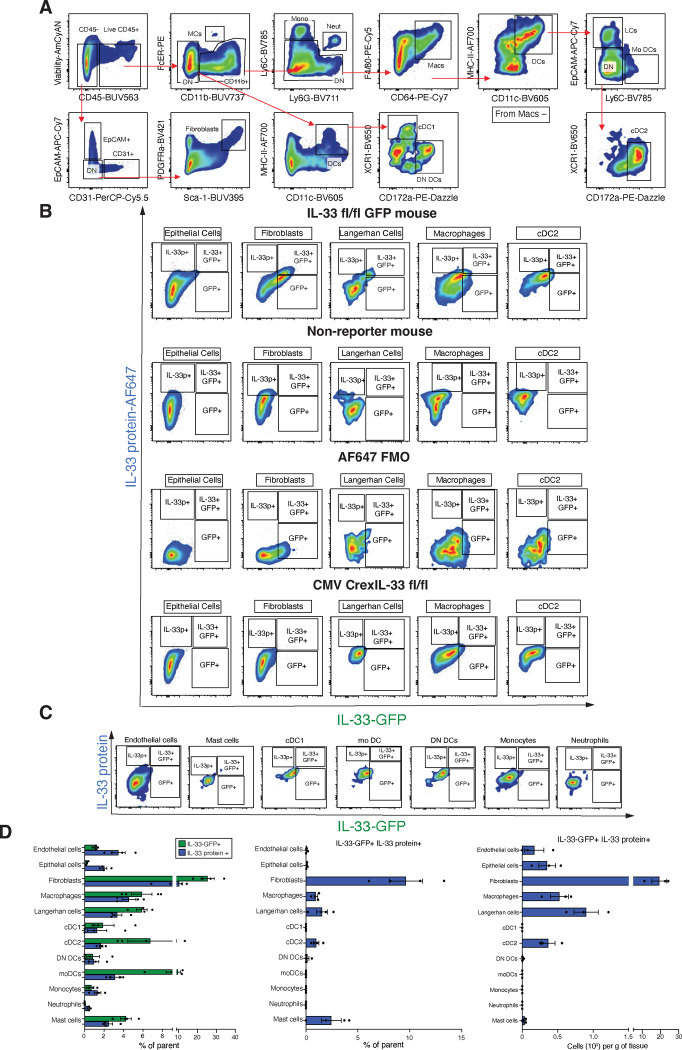
Dermal myeloid cells express IL-33. (**A**), Flow cytometric gating strategy to analyze hematopoietic and non-hematopoietic cell subsets from abdominal skin explants from naïve IL-33^fl/fl^ IRES-eGFP mice. (**B,C**), Representative flow cytometric plots of IL-33 protein and IL-33-GFP transcript expression within specific subsets of skin-resident cells from IL-33fl/fl GFP mice as well as staining controls. (**D**), Quantification of percentages and absolute numbers of IL-33-GFP+, IL-33-protein+, and IL-33-GFP+ IL-33protein+ cutaneous cells.

**Extended Data Figure 4.**
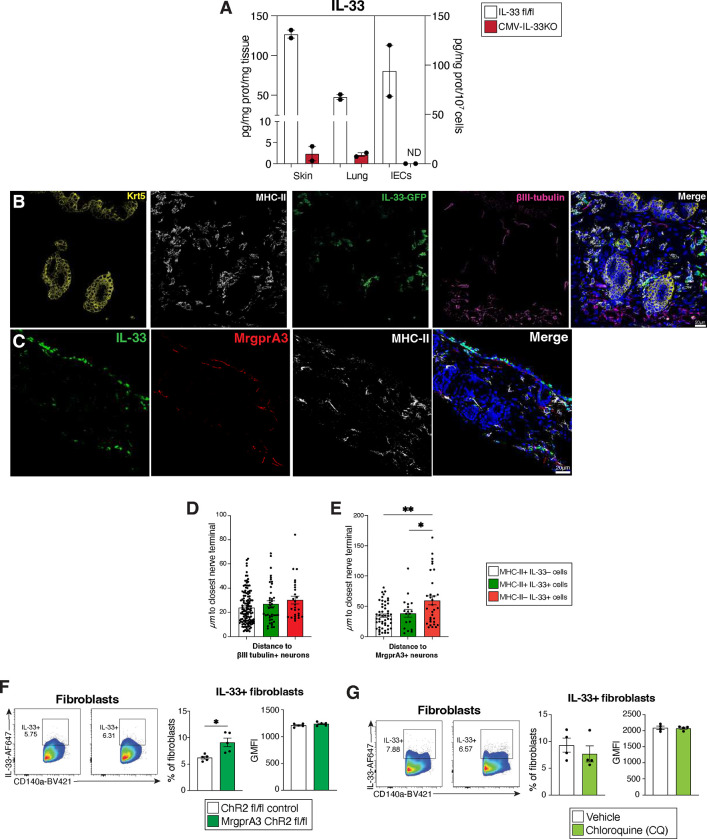
Skin-resident myeloid APCs localize closely to MrgprA3+ neurons. (**A**), Tissue and cell IL-33 protein levels were quantified in CMV-IL-33KO mice. (**B**), Individual channels of Immunofluorescence (IFA) staining for Keratin 5 (Krt5), MHC-II, βIII tubulin and IL-33-GFP in tissue sections from naïve abdominal skin of IL-33^fl/fl^ IRES-eGFP mice. (**C**), Individual channels of MHC-II, MrgprA3, and IL-33 in naïve abdominal skin sections of MrgprA3^Cre+/−^:Rosa26^tdTomato^ mice. (**D,E**), Quantification of the distance between IL-33+, MHC-II+ or IL-33+ MHC-II+ cells with respect of βIII tubulin+ or MrgprA3+ neurons. (**F,G**), Quantification of skin IL-33+ fibroblasts of MrgprA3-ChR2 or CQ-treated mice compared with their respective controls. *P* values were determined by two-tailed Student’s t-tests or One-way ANOVA with post hoc correction. *P<0.05, **P<0.01. Representative of 2 independent experiments, each with 3 biological replicates.

Although IL-33 is widely held as a nuclear cytokine expressed by structural cells, such as epithelial and endothelial cells^26^, we and others have demonstrated that myeloid cells can basally express and release IL-33 to regulate mucosal inflammation^27, 28, 29^. To investigate whether MrgprA3 neurons could also regulate myeloid IL-33 expression, we first asked whether myeloid cells in the skin basally expressed IL-33. Using IL-33^fl/fl^ IRES-eGFP mice that allow tracking of *Il33* transcript expression^27, 44^, we used a combination of transcript and protein content evaluation using intracellular flow cytometry to account for potential post-transcriptional differences. IL-33 levels were evaluated in both hematopoietic and non-hematopoietic populations within naïve skin, including epithelial cells, endothelial cells, fibroblasts, macrophages, Langerhan cells (LCs), type 1 conventional dendritic cells (cDC1), cDC2, monocyte-derived dendritic cells (moDCs), monocytes, neutrophils, and mast cells (Extended Data Fig.3a). For validation of this combined approach involving IL-33-GFP and intracellular IL-33 protein content, our negative controls included WT C57BL/6 non-reporter mice, FMO controls, and mice generated by crossing the IL-33^fl/fl^ IRES-eGFP with the CMV-Cre strain^45^, resulting in broad deletion of IL-33 expression in skin, lung and intestinal epithelial cells (Extended Data Fig.3b, 4a).

**Figure 3.**
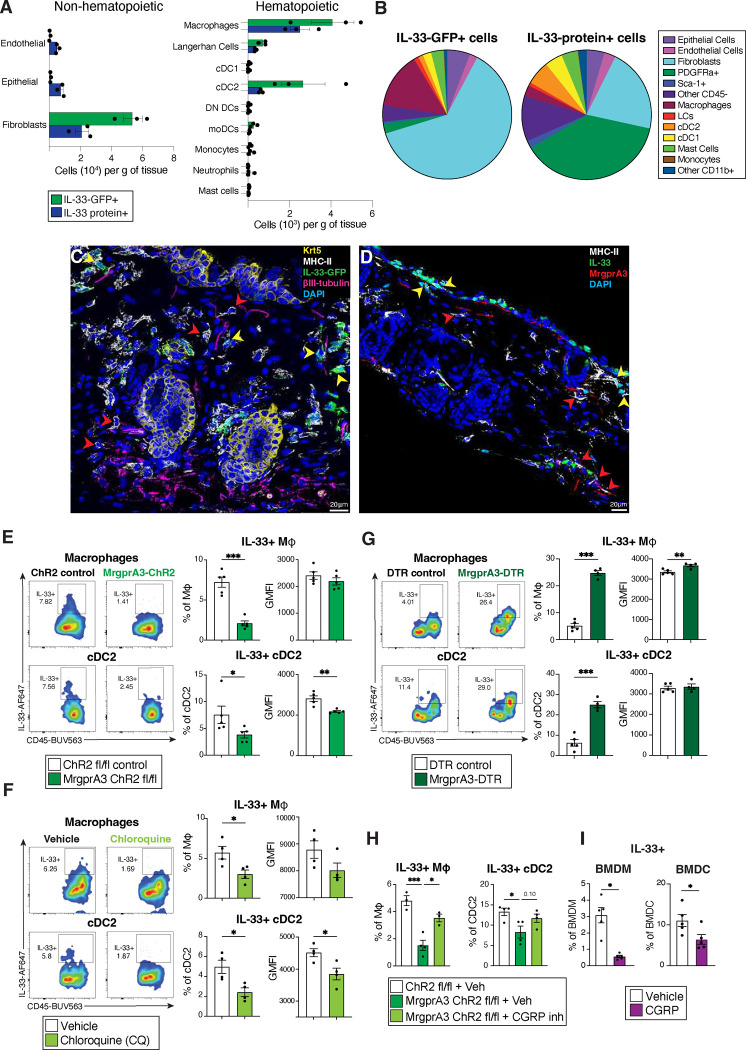
MrgprA3 neurons control IL-33 expression in skin myeloid antigen presenting cells (APC). (**A**), Quantification of absolute cell numbers of IL-33-GFP+ and IL-33-protein+ non-hematopoietic and hematopoietic cells in unmanipulated skin of IL-33^fl/fl^ IRES-eGFP mice. (**B**), Proportions of cutaneous cell populations defined by IL-33-GFP or IL-33-protein expressions. (**C**), Immunofluorescence (IFA) staining for Keratin 5 (Krt5), MHC-II, βIII tubulin, and IL-33-GFP in tissue sections from naïve abdominal skin of IL-33^fl/fl^ IRES-eGFP mice. Red arrows indicate IL-33- MHC-II+ cells, yellow arrows indicate L-33+ MHC-II+ cells. (**D**), MHC-II, MrgprA3, and IL-33 in naïve abdominal skin sections of MrgprA3^Cre+/−^:Rosa26^tdTomato^ mice. Red arrows indicate IL-33-MHC-II+ cells, yellow arrows indicate L-33+ MHC-II+ cells. (**E**), Representative plots and quantification of IL-33+ frequencies and GMFI in tissue macrophages and cDC2 from light stimulated MrgprA3-ChR2 mice or CQ-injected wildtype mice 5 days post-treatment. (**F**), Skin IL-33+ macrophages and cDC2 were quantified by flow cytometry in vehicle or CQ-treated mice 5 days post-treatment. (**G**), IL-33 expression in myeloid APCs was quantified by flow cytometry of control or MrgprA3-DTR mice after 3 weeks of DT systemic administration. (**H**), IL-33+ macrophages and cDC2s from control, vehicle-treated MrgprA3-ChR2, and CGRP inhibitor-treated MrgprA3-ChR2 mice were quantified by flow cytometry 5 days post-optogenetic stimulation. (**I**), IL-33 protein expression in bone marrow-derived macrophages (BMDMs) and dendritic cells (BMDCs) was evaluated 24hrs. after treatment with recombinant CGRP. *P* values were determined by two-tailed Student’s t-tests, One-way ANOVA or Two-way ANOVA with post hoc correction. *P<0.05, **P<0.01, ***P<0.001, ****P<0.0001. Representative of 2–3 independent experiments, each with ≥4 biological replicates.

Among non-hematopoietic cells, skin fibroblasts expressed highest levels of IL-33 protein and transcript, (Fig. 3a, Extended Data Fig.3b–d), consistent with a previous report^46^. Endothelial and epithelial cells contained moderate levels of IL-33 protein and low transcript abundance (Fig. 3A\a, Extended Data Fig.3b–d). Importantly, among hematopoietic cells, both IL-33 transcript and protein was detectable in LCs, macrophages, cDC2s, and mast cells, but not in neutrophils, monocytes or cDC1s (Fig. 3a and Extended Data Fig.3b–d). Of note, CD45+ CD11b+ myeloid cell populations represented approximately 20% of all IL-33-GFP+ or IL-33 protein-expressing cells in the skin, with macrophages being the most prevalent IL-33-GFP+ population of all CD45+ cells (Fig. 3b).

Next, to determine whether IL-33 expressing skin-resident antigen presenting cells (APC) were positioned near neurons populations, we assessed the proximity of MHC-II+ cells, either with or without IL-33 GFP expression, to βIII-tubulin+ sensory afferents in skin. There were no significant differences in the distance between IL-33+ or IL-33− MHC-II+ antigen presenting cells (APCs) and non-APC IL-33+ to nerve terminals in general (Fig. 3c, Extended Data Fig.4b,d). In contrast, using MrgprA3-tDtomato reporter mice to assess the proximity of IL-33+ MHC-II+ cells to itch-transmitting neurons ^11, 32^ revealed that MrgprA3+ afferents were more closely associated with MHCII+ IL-33− cells than MHCII- IL-33+ cells in naïve skin (Fig. 3d and Extended Data Fig.4c,e).

Given this association between MrgprA3 neurons and IL-33 expressing skin APC, we tested whether these neurons could modulate IL-33 expression. Surprisingly, optogenetic stimulation of MrgprA3+ neurons significantly reduced the frequency and IL-33 fluorescence intensity of tissue macrophages and cDC2 containing IL-33 protein; an effect not observed on IL33+ fibroblasts (Fig. 3e; Extended Data Fig.4f). MrgprA3 neuron activation following CQ administration also significantly reduced the frequency of APCs containing IL-33 protein, but also did not significantly change IL-33 expression in fibroblasts (Fig. 3f, Extended Data Fig.4g). Consistent with these data, MrgprA3 neuron ablation significantly increased the frequency of skin macrophages and cDC2 containing IL-33 protein (Fig. 3g). To determine if CGRP was required to downregulate IL-33 expression in APCs, mice subjected to optogenetic activation of MrgprA3 neurons were given the CGRP inhibitor CGRP_8–37_, which restored IL-33 expression in macrophages and cDC2 (Fig. 3h). Moreover, rCGRP alone was sufficient to downregulate IL-33 expression in myeloid APC, shown by exposure of BMDM or BMDC cultures to exogenous CGRP (Fig. 3i). These data collectively suggest that, in contrast to induction of IL-1β and TNFα, activated MrgprA3 neurons downregulated the ability of myeloid APC to express IL-33, at least in part, via the neuropeptide CGRP.

### Myeloid-specific IL-33 restrains IL-17+ cutaneous responses that promote keratinization

**Extended Data Figure 5.**
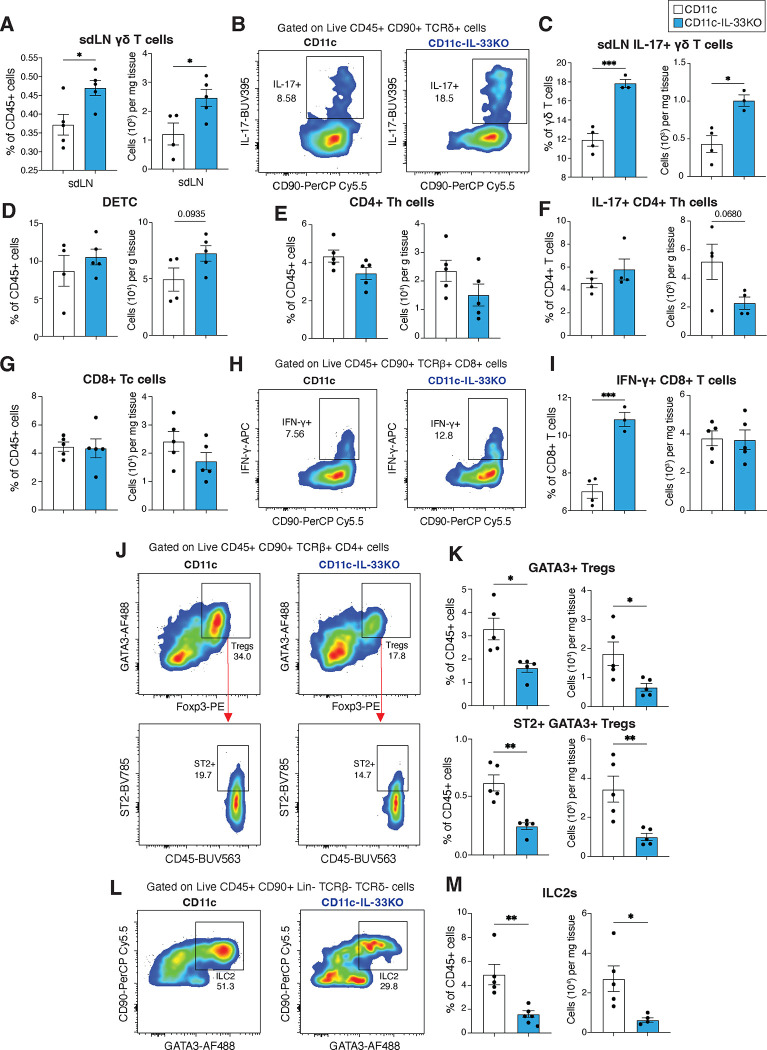
IL-33 expression in APCs limits IL-17 cutaneous responses and sustain dermal T regulatory cells and ILC2s. (**A-C**), Representative plots and quantification of γδ T cells and IL-17+ γδ T cells in skin-draining lymph nodes (sdLNs) from control or CD11c-IL-33KO mice. Naïve abdominal skin from control or CD11-IL-33KO mice was evaluated for (**D**), DETC, (**E,F**), CD4+ T helper and IL-17+ CD4+ T cells, (**G-I**), CD8+ cytotoxic T cells and IFNγ+ CD8+ T cells, (**J,K**), ST2+ GATA3+ T regulatory cells, and (**L,M**), GATA3+ ILC2 cell populations. *P* values were determined by two-tailed Student’s t-test. *P<0.05, **P<0.01, ***P<0.001. Representative of 3 independent experiments, each with ≥4 biological replicates.

**Figure 4.**
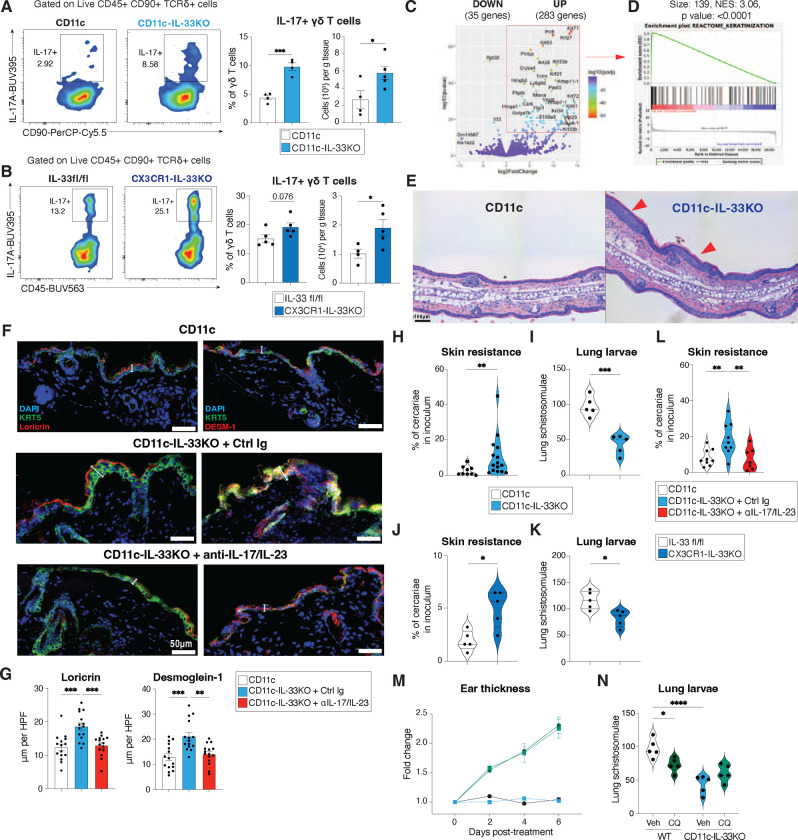
Myeloid-derived IL-33 maintains skin homeostasis by limiting IL-17-driven excessive keratinization. (**A,B**), Representative plots and quantification of dermal IL-17+ γδ T cell responses from CD11c-IL-33KO or CX3CR1-IL-33KO mice. (**C**), Volcano plots of bulk RNA-seq analysis of differentially expressed genes from naïve abdominal skin explants from control or CD11c-IL-33KO mice. (**D**), GSEA enrichment plots of pathway analysis in genes enriched in naïve skin from CD11c-IL-33KO mice. (**E**), FFPE ear skin sections from naïve control or CD11c-IL-33KO mice were analyzed by H&E staining. (**F**), Immunofluorescence (IFA) staining for Loricrin or Desmoglein-1 (Desm-1) in skin sections from control, CD11c-IL-33KO, or CD11c-IL-33KO treated with neutralizing antibodies against IL-17 and IL-23. White bars represent epidermal thickness. (**G**), Quantification of epidermal thickening in Loricrin- or Desm-1-stained skin sections in control, CD11c-IL-33KO, CD11c-IL-33KO mice treated with neutralizing antibodies against IL-17 and IL-23. (**H,I**), Percentage of cercariae remaining in the inoculum and D6 lung larval burden of CD11c-IL-33KO or (**J,K**), CX3CR1-IL-33KO mice and their respective controls after *S. mansoni* cutaneous exposure. (**L**), CD11c-IL-33KO mice were treated with neutralizing antibodies against IL-17 and IL-23 for 10 days prior to cercarial ear exposure. The proportion of non-penetrating cercariae was compared to CD11c control or Ig-treated (Ctrl Ig) CD11c-IL-33KO mice. (**M,N**), Ear thickness and D6 *S. mansoni* lung larval burden of control and CD11c-IL-33KO mice treated with vehicle or MrgprA3 agonist, chloroquine (CQ) prior to infection. *P* values were determined by two-tailed Student’s t-tests, One-way ANOVA, or Two-way ANOVA with post hoc correction. *P<0.05, **P<0.01, ***P<0.001, ****P<0.0001. Representative of 2–3 independent experiments, each with ≥4 biological replicates.

It is known that skin-resident APCs can promote keratinocyte turnover and effector function(s) via secreting cytokines such as IL-1β, TNFα, and IL-23^37, 38, 47^. Given the apparent inverse relationship between IL-33 and these IL-17 promoting cytokines (e.g., IL-1β, TNFα) we directly tested whether a selective deletion of IL-33 in discrete populations of myeloid-APC would impact skin architecture or immune cell content. Skin biopsies of naïve IL-33^fl/fl^xCD11c^Cre^ (CD11c-IL-33KO) or IL-33^fl/l^xCX3CR1^cre^ (CX3CR1-IL-33KO) mouse strains were compared to their respective co-housed littermate controls under steady-state conditions. In both cases, myeloid-specific IL-33 deletion basally increased the frequency of IL-17+ γδ T cells as determined by flow cytometry (Fig. 4a,b), which was also reflected in the skin-draining lymph nodes (sdLNs) of CD11c-IL-33KO (Extended Data Fig.5a–c). To investigate whether IL-33-expressing myeloid cells regulated the abundance or activation state of other skin resident lymphocyte populations, flow cytometry was used to evaluate DETC, CD4+ Th, and CD8+ T cell number, but loss of IL-33 in CD11c+ cells did not result in significant changes in these populations (Extended Data Fig.5d–g). However, deficiency in myeloid derived IL-33 did increase the proportion of IFN-γ+ CD8 T cells, significantly reduced both the number and frequency of both GATA3+ Tregs and ST2+ GATA3+ Tregs, and significantly diminished ILC2s as compared to CD11c controls (Extended Data Fig.5h–m). Together, these results suggested that IL-33-expressing myeloid cells regulated the balance between γδ T cells and GATA3+ Tregs and ILC2s in the skin.

**Extended Data Figure 6.**
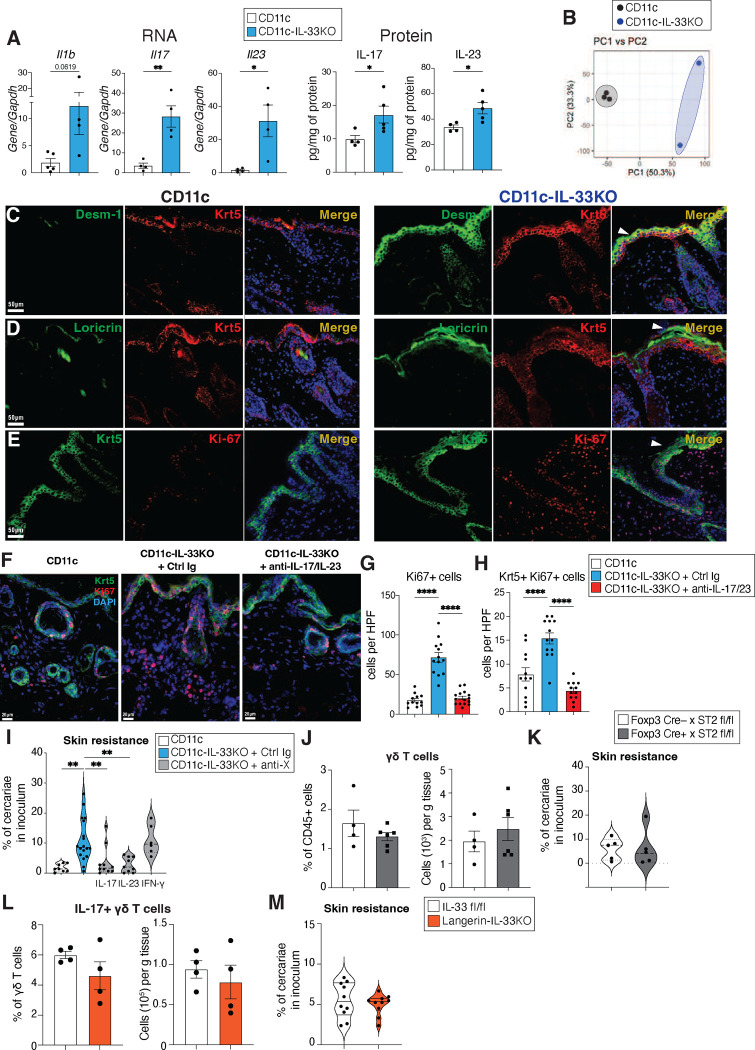
Myeloid-derived IL-33 limits IL-17-driven cutaneous immunity. (**A**), RNA and protein levels of cytokines from naïve skin explants of control or CD11c-IL-33KO mice. (**B**), Principal component analysis (PCA) of bulk RNA-seq analysis of naïve abdominal skin explants from control or CD11c-IL-33KO mice. (**C-E**), Individual channels of IFA staining for Loricrin, Desmoglein-1 (Desm-1), or Ki-67 in skin sections from control or CD11c-IL-33KO mice. White arrows indicate accumulation of these proteins. (**F**), IFA staining for Ki-67 in skin sections from control, CD11c-IL-33KO, or CD11c-IL-33KO treated with neutralizing antibodies against IL-17 and IL-23. (**G,H**), Quantification of Ki-67+ and Krt5+ Ki-67+ cells in skin sections of control, CD11c-IL-33KO, or CD11c-IL-33KO treated with neutralizing antibodies against IL-17 and IL-23. (**I**), CD11c-IL-33KO mice were treated with neutralizing antibodies against IL-17, IL-23, or IFNγ for 10 days prior to cercarial abdominal exposure. The proportion of non-penetrating cercariae was compared to CD11c control or Ig-treated (Ctrl Ig) CD11c-IL-33KO mice. (**J,K**), Dermal γδ T cells and percentage of non-penetrating cercariae were quantified in Foxp3Cre :ST2fl/fl mice. (**L,M**), IL-17+ dermal γδ T cells and percentage of non-penetrating cercariae were quantified in Langering Cre-IL-33KO mice. *P* values were determined by two-tailed Student’s t-test or Oneway ANOVA with post hoc correction. *P<0.05, **P<0.01, ***P<0.001. Representative of 3 independent experiments, each with ≥4 biological replicates.

Congruent with altered immune cell content, the skin of CD11c-IL-33KO mice also had elevated transcript levels of *Il1b*, and elevated transcript and protein levels of IL-17A, and IL-23 (Extended Data Fig.6a). Bulk RNA sequencing of abdominal skin from naïve CD11c-IL-33KO mice presented with a distinct transcriptional profile compared to littermate control skin in the CD11c^Cre^ strain (CD11c) (Fig. 3c, Extended Data Fig.6b). Gene set enrichment analysis (GSEA) revealed an over-representation of gene pathways associated with keratinization in CD11c-IL-33KO mice as compared to CD11c littermate controls (Fig. 3d). H&E stained FFPE sections of ear skin revealed that CD11c-IL-33KO had increased epidermal hyperplasia and compact hyperkeratosis when compared to CD11c controls (Fig. 3e). To investigate whether these anatomical changes reflected distinct alterations in the epidermis, we evaluated both Loricrin expression, a cornified envelope protein, and Desmoglein-1, a keratinocyte maturation marker ^2^. CD11c-IL-33KO mice presented with elevated epidermal accumulation of Loricrin and Desmoglein-1 apically to Keratin-5 (Krt5)-expressing basal keratinocytes (Extended Data Fig.6c,d). Immunostaining with the S-phase marker Ki-67 revealed a marked increase in the number of proliferating basal keratinocytes in the epidermis and dermal cells due to myeloid-specific IL-33 deficiency (Extended Data Fig.6e). Thus, even under basal conditions, the loss of myeloid-specific IL-33 deficiency markedly changed skin architecture.

To directly assess whether IL-17 dominated inflammation was responsible for promoting the hyperkeratinization in mice lacking IL-33 in CD11c+ cells, neutralizing antibodies for IL-17A and IL-23 were administered to CD11c-IL-33KO mice. Over the course of 5 days, treatment with this Ab cocktail reversed the epidermal thickness and decreased the number of proliferating Ki67+ cells CD11c-IL-33KO mice (Fig. 4f,g; Extended Data Fig.6f–h). To test the biological importance of these IL-17- dependent alterations to skin architecture due to loss of myeloid IL-33, CD11c-IL-33KO and CX3CR1-IL-33KO strains were subjected to *S. mansoni* infection. Both strains exhibited increased resistance to skin penetration by *S. mansoni* cercariae and reduced larval burdens in lung tissue at 6 days post-infection (Fig. 4h–k). Neutralization of IL-17 and IL-23, but not IFN-γ reversed the skin resistance of CD11c-IL-33KO mice to *S. mansoni* (Fig. 4l, Extended Data Fig.6i).

Next, we turned our focus to potential causes of this enhanced IL-17 inflammation and investigated whether loss of ST2+ Tregs or deletion of IL-33 in Langerin+ cells phenocopied the skin alterations in CD11c-IL-33KO mice. First, ST2fl/fl mice were interbred with a Foxp3Cre expressing strain given previous data implicating IL-33 responsive Tregs in regulating γδ T cells ^48^. However, this strain did not develop altered dermal γδ T cells or additional protection against *S. mansoni* skin entry (Extended Data Fig.6j,k). Similarly, genetic deletion of IL-33 in Langerin-expressing cells ^49^, that include LCs and skin cDC1^1^, did not phenocopy the alterations of γδ T cells or susceptibility to *S. mansoni* in CD11c-IL-33KO mice (Extended Data Fig.6l,m). This suggested that disruption of certain myeloid-derived IL-33 subsets (CD11c+ or CX3CR1+), but not others (i.e., Langerin-expressing APC) promoted IL-17/IL-23 cytokine responses, driving excessive keratinization and cutaneous anti-helminth immunity under basal conditions.

Lastly, to evaluate the specific interaction between MrgprA3 neuron activation and the presence of IL-33 in myeloid cells, we treated CD11c-IL-33KO and controls with CQ, and found that these mice presented with similar levels of ear swelling and lung larval burdens following infection with *S. mansoni* cercariae (Fig. 4m,n), indicating that the effects of MrgprA3 neuron activation on skin inflammation and host protection primarily relied upon downregulation of myeloid-specific IL-33 and could not be induced in myeloid cells genetically lacking IL-33. Thus, loss of myeloid-specific IL-33 alone set in motion alterations that promoted an IL-17 dependent anthelminthic skin environment.

### IL-33 regulates pro-inflammatory cytokine secretion by myeloid cells

**Extended Data Figure 7.**
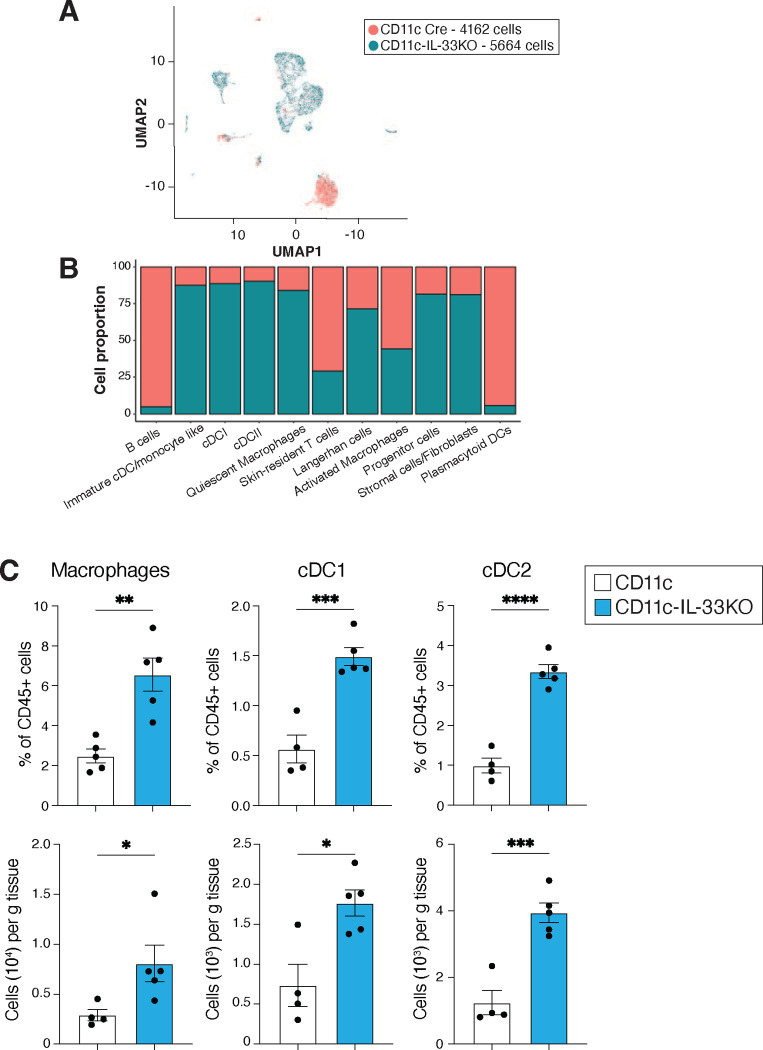
Ablation of IL-33 expands myeloid cell populations in the skin at baseline. (**A,B**), UMAP plots and quantification of cell proportion from scRNA-seq cell clusters in control or CD11c-IL-33KO mice. (**C**), Quantification of macrophages, cDC1s, and cDC2s in naïve skin from control or CD11c-IL-33KO mice by flow cytometry. *P* values were determined by two-tailed Student’s t-test. *P<0.05, **P<0.01, ***P<0.001. Representative of 2–3 independent experiments, each with ≥4 biological replicates.

**Extended Data Figure 8.**
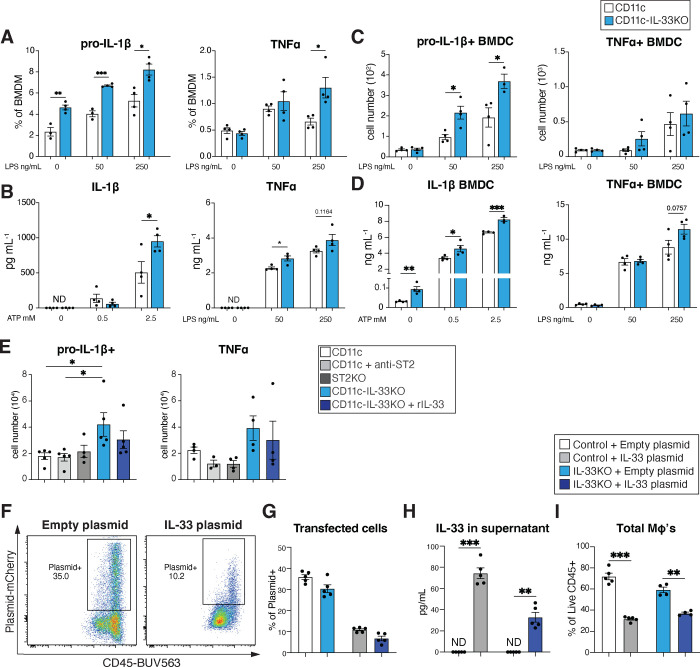
IL-33 intrinsically limits cytokine secretion by myeloid cells. **(A,B)**, Percentage of cytokine+ and cytokine levels in supernatant from control or CD11c-IL-33KO BMDMs treated with increasing concentrations of LPS or 250ng/mL of LPS followed by ATP stimulation for mature IL-1β release. **(C,D)**, Cytokine-expressing cells and levels in supernatant from control or CD11c-IL-33KO bone marrow-derived dendritic cells (BMDCs) treated with increasing concentrations of LPS or 250ng/mL of LPS followed by ATP stimulation for mature IL1-β release. **(E)**, LPS-induced cytokine-expressing cells from CD11c control, anti-ST2-treated control, ST2-deficient, CD11c-IL-33KO, or rIL-33-treated CD11c-IL-33KO BMDMs. **(F,G)**, Representative counterplots and quantification of plasmid+ control or CMV-IL-33KO BMDMs 2 days post-transfection with empty or IL-33-containing plasmid. **(H)**, IL-33 supernatant levels from control or CMV-IL-33KO BMDMs transfected with empty- or IL-33-containing plasmid. **(I)**, Total macrophages were quantified in control or CMV-IL-33KO BMDMs that were transfected with empty or IL-33-containing plasmid. *P* values were determined by two-tailed Student’s t-tests or One-way ANOVA with post hoc correction. *P<0.05, **P<0.01. ***P<0.001. C. Representative of 2–3 independent experiments, each with ≥4 biological replicates.

**Figure 5.**
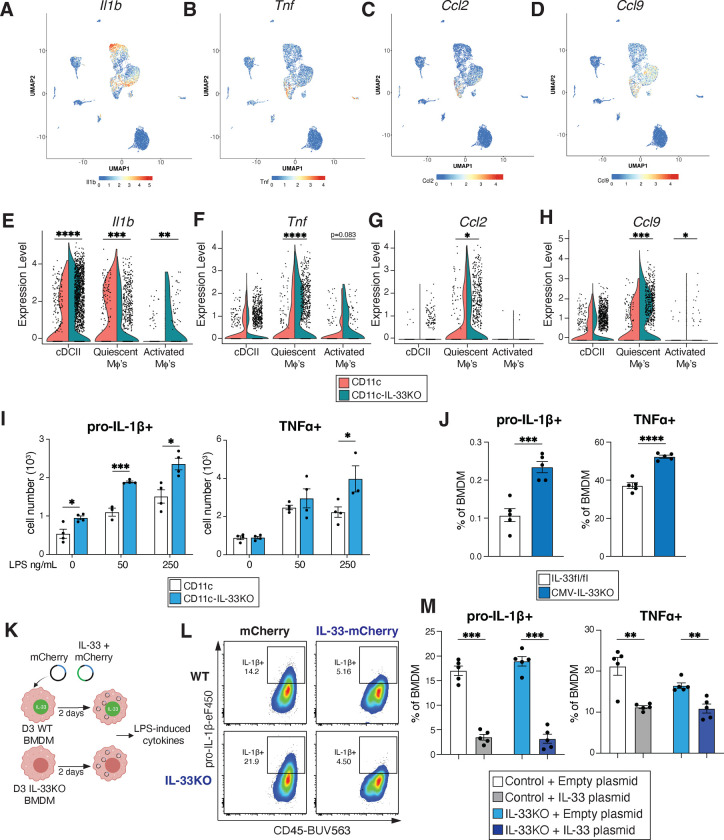
IL-33 intrinsically regulates cytokine expression in cutaneous myeloid cells. (**A-D**), UMAP plots illustrating cytokine and chemokine gene expression among myeloid cell clusters generated by single cell RNA-sequencing of skin-resident CD11c+ MHC-II+ populations sort-purified from unmanipulated control or CD11c-IL-33KO mice. (**E-H**), Violin plots of cytokine and chemokine gene expression in specific myeloid APC clusters from control or CD11c-IL-33KO skin. (**I**), Cytokine-expressing cells from control or CD11c-IL-33KO BMDMs treated with increasing concentrations of LPS. (**J**), LPS-induced cytokine expression of BMDMs generated from control or CMV-IL-33KO mice. (**K**), Experimental approach to transfect control or CMV-IL-33KO BMDMs with empty mCherry plasmid or IL-33-mCherry plasmid followed by quantification of LPS-induced cytokines 2 days post-transfection. (**L,M**), Representative counterplots of pro-IL-1β staining and quantification of pro-IL-1β+ and TNFα+ cells in control or CMV-IL-33KO BMDMs that were transfected with empty or IL-33-containing plasmid. *P* values were determined by two-tailed Student’s t-tests or One-way ANOVA with post hoc correction. *P<0.05, **P<0.01, ***P<0.001. I-M, Representative of 2–3 independent experiments, each with ≥4 biological replicates.

To further investigate the importance of myeloid-specific IL-33 as a regulator of IL-17-driven inflammation in the skin, single cell RNA sequencing was done on sort purified CD11c+ MHCII+ cells from the skin of unmanipulated CD11c control and CD11c-IL-33KO mice (Fig. 2h, Extended Data Fig.7a). Macrophage and dendritic cell subsets were expanded in mice lacking myeloid IL-33 as determined by scRNAseq and flow cytometry (Extended Data Fig.7b,c). cDC2, quiescent and activated macrophage clusters presented with significant enrichment for the proinflammatory cytokines *Il1b, Tnf,* which are known regulators of skin-lymphocyte responses ^3, 50, 51, 52^ and the chemokines *Ccl2 and 9*, which promote dermal γδ T cell responses ^53^ and myeloid-cell recruitment^54, 55^ respectively (Fig. 5a–d). While cDC2’s exhibited increased *Il1b* expression, macrophage clusters presented with enhanced *Tnf, Ccl2,* and *Ccl9* expression (Fig. 5e–h). These cell-intrinsic transcriptional changes in cytokine and chemokine expression due to *in vivo* conditional deletion of IL-33 prompted further investigation using BMDMs cultured from CD11c-IL-33KO mice or controls. Again, lack of IL-33 was associated with enhanced pro-IL1β, and TNFα expression and release evoked by LPS (Fig. 5i and Extended Data Fig.8a,b), which was also true for pro-IL1β in BMDCs and BMDMs derived from CMV-IL-33KO mice (Fig. 5j, Extended Data Fig.8c,d). Because it remained possible that secreted IL-33 acting on its receptor ST2 could potentially explain these effects, BMDM culture experiments were conducted that used either antibody-mediated neutralization of ST2 under wild-type conditions, genetic loss of ST2, or exogenous rIL-33 treatment of IL-33 deficient myeloid cells. None of these conditions rescued the alterations in LPS-induced cytokine expression observed due to cell intrinsic IL-33 deficiency (Extended Data Fig.8e). Thus, we tested if overexpression of cell-intrinsic IL-33 impaired pro-inflammatory cytokine expression in control and IL-33KO BMDMs transfected with an IL-33-mCherry-expressing plasmid as compared to empty vector transfection followed by LPS stimulation (Fig. 5k). We confirmed that both approaches resulted in detectable mCherry expression, albeit IL-33-plasmid transfection occurred at lower efficiency, but this was still sufficient to result in detection of soluble IL-33 from both WT and IL-33KO cells (Extended Data Fig.8f–h). Further, IL-33 transfection led to suppression of LPS-induced pro-IL1β and TNFα expression (Fig. 5l and 5m). IL-33 overexpression resulted in reduced viability irrespective of BMDM genotype (Extended Data Fig.8i), perhaps related to the expansion of multiple myeloid populations in the absence of IL-33 *in vivo*. These findings further supported a hypothesis that IL-33 could potentially suppress pro-inflammatory cytokine expression in a myeloid cell intrinsic manner.

### Myeloid cell intrinsic IL-33 alters chromatin accessibility

**Figure 6.**
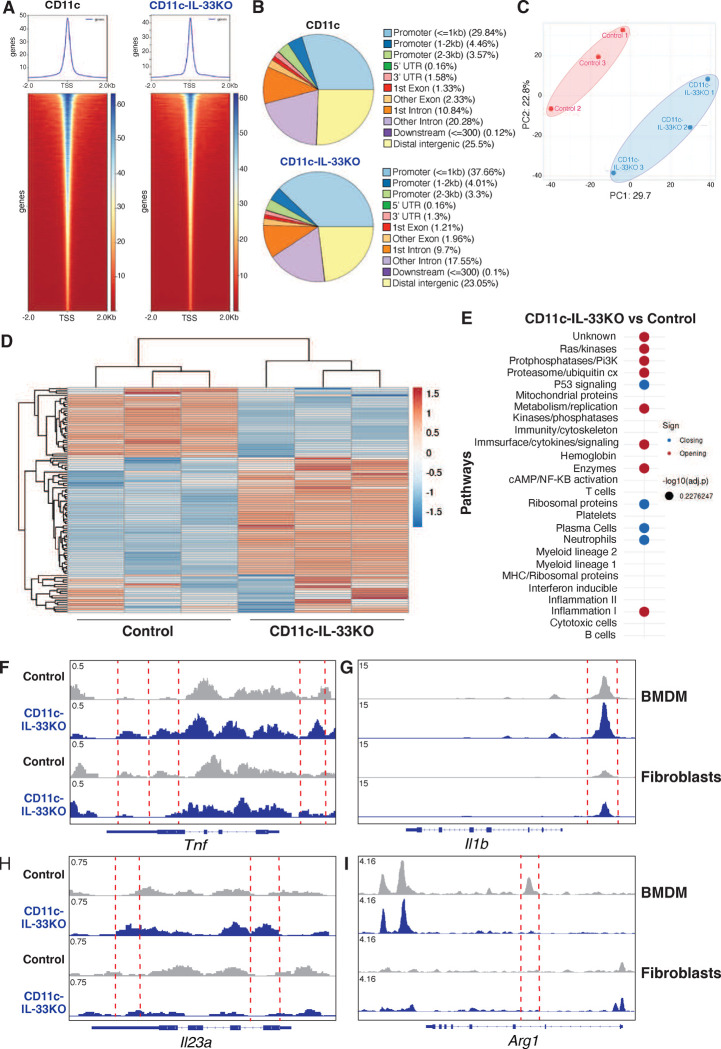
IL-33 is required to control chromatin accessibility at pro-inflammatory gene loci in macrophages. (**A**), Heatmap showing distance of accessible regions to transcriptional start sites (TSS) of control or CD11c-IL-33KO bone-marrow derived macrophages (BMDMs). (**B**), Genomic features of open chromatin regions in control or CD11c-IL-33KO BMDMs. (**C,D**), Principal component analysis (PCA) and heatmaps of accessible chromatin regions in control or CD11c-IL-33KO BMDMs. (**E**), Hypergeometric p-value (HPEA) analysis of functional enrichment in accessible regions of CD11c control or CD11c-IL-33KO BMDMs. (**F-I**), Genome browser views of distinct accessible regions (boxed) in *Tnf, Il1b, Il23a,* and *Arg1* loci of control or CD11c-IL-33KO BMDMs or fibroblasts. Representative of 2 independent experiments, each with 3 biological replicates.

Whether IL-33 can intrinsically control transcriptional activity is highly controversial, despite its well-established localization to the nucleosome acidic patch comprised of the tails of histones H2A and H2B^56^. Bulk ATAC-sequencing was used to determine whether chromatin accessibility was altered by myeloid-intrinsic loss of IL-33. Surprisingly, loss of IL-33 in BMDMs resulted in fewer open regions at transcriptional start sites (TSS) compared to CD11c controls (Fig. 6a). Overall peak distribution by genomic features revealed a change in distribution of open chromatin regions between groups, such that 29.8% of peaks map to ≤1kb promoters in CD11c BMDM, vs ~37.7% of peaks map to such promoters in CD11c-IL-33KO BMDM. Conversely there was a relative increase in intron and distal intergenic peaks in CD11c-IL-33KO BMDM as compared to CD11c control BMDM (Fig. 6b). Principal component analysis revealed that control and CD11c-IL-33KO BMDMs differentially clustered and presented with distinct chromatin accessibility profiles (Fig. 6c,d). Hypergeometric p-value (HPEA) analysis of functional enrichment revealed that CD11c-IL33KO presented with open regions related to Ras/kinases, proteasome/ubiquitination, metabolism/replication, immune surface/cytokine signaling and Inflammation (Fig. 6e). Peak calling of specific genes showed that CD11c-IL-33KO BMDMs had increased chromatin opening within coding regions of the *Il23a* and *Tnf* genes compared to control BMDMs (Fig. 6f,h). Further, peak calling of CD11c-IL-33KO BMDMs at the *Il1b* locus showed moderately enhanced accessibility at a region approximately 2kb upstream of its TSS previously reported to be an enhancer^57^ (Fig. 6g). Conversely, the *Arg1* locus showed markedly reduced accessibility in intergenic regions of IL-33 deficient BMDMs (Fig. 6i). These alterations in chromatin accessibility of specific loci were not observed in fibroblasts derived from CD11c-IL-33KO mice (Fig. 6f–i). Taken together, these data suggest that genetic deficiency in IL-33 altered the chromatin accessibility of distinct pro-inflammatory and anti-inflammatory genes expressed in myeloid cells.

## Discussion

Optimal maintenance of organismal health under steady-state and infection requires delicately orchestrated processes involving diverse cell types and soluble mediators. Such cellular crosstalk involves mechanisms for rapid sensing/detection, initiation of inflammation, and response termination for restoration of homeostasis. Herein, we provide evidence that MrgrpA3+ skin sensory neurons function to regulate cytokine expression patterns in myeloid APC that promote cutaneous inflammation and anti-helminth resistance. When MrgprA3 neurons are stimulated, they release neuropeptides such as CGRP, that can signal through cognate receptors expressed on tissue-resident myeloid subsets. CGRP signaling in macrophages alters cytokine expression patterns, namely to reduce IL-33. Activation of MrgprA3 neurons downregulates intracellular IL-33 protein content in macrophages and cDC2, partially through CGRP, and the sole loss of IL-33 is alone sufficient to increase IL-17+ γδ T cell responses and resistance to *S. mansoni* skin invasion. Given our data showing that IL-33 regulated chromatin accessibility at loci encoding pro-inflammatory cytokine genes, we speculate that the ability of itch neurons to downmodulate IL-33 allows macrophages and cDC2s to revert from a quiescent state to release pro-inflammatory cytokines such as IL-1β, TNFα, and IL-23 for stimulation of IL-17-producing γδ T cells^37, 38, 47^. Functionally, this induction of IL-23/IL-17-mediated inflammation reshapes the skin environment through driving keratinocyte turnover, neutrophil recruitment, and accumulation of skin barrier proteins like Desmoglein-1 and Loricin at the uppermost layers of the stratified epithelium. Such remodeling of the skin hinders penetration by infectious larval stages of *S. mansoni* and further enables potent host- responses that limit parasite migration to other tissues. Overall, these data further emphasize the importance of myeloid-intrinsic IL-33 in regulating the balance between pro vs. anti-inflammatory responses at barrier surfaces and reveals that such mechanisms can be controlled by the somatosensory nervous system.

A growing body of literature has recognized host-protective skin immunity against viral, bacterial, and fungal pathogens. Surprisingly, however, little is known about the cellular and molecular events that occur upon the natural process of skin entry by helminth parasites such as hookworms or trematode species (*Schistosoma spp.*), and whether these events are effective at preventing helminth entry or migration. TRPV1+ neurons have been previously shown to confer anticipatory immunity against fungal pathogens that invade the epidermis of the skin^12, 13^. Although some MrgprA3 neurons express TRPV1^11, 32^ and may also have been targeted in these previous studies, whether discrete subsets of pruriceptors control immunity to skin-penetrating helminths was previously unaddressed. Although controlled experimental exposure to *S. mansoni* causes mild dermatitis and pruritus^58^, most natural infections go unnoticed by the host. By contrast, human cutaneous infection with bird schistosomes often result in intense pruritic episodes known as ‘Swimmers itch’^59^, but these cross-species infections are often abortive and do not disseminate systemically. Our data showing that activation of itch-inducing MrgprA3+ neurons limits cercarial penetration and enhances anti-schistosome immunity makes it tempting to speculate that neuronal-myeloid crosstalk may limit patent infection of humans with avian schistosomes. By extension, it is possible that human schistosome species may have evolved neuro-suppressive factors as a countermeasure that limit the activation of itch-mediating neurons and subsequent cutaneous inflammation to facilitate host parasitism, which could result in less perceived itch and scratching behavior. This prediction is further supported by studies revealing how bacterial pathogens can activate TRPV1+ neurons to suppress immune responses that would counter their cutaneous dissemination^60, 61^.

Our findings support other recent studies linking TRPV1+ nociceptor activation to IL-17 driven cutaneous immunity but identify the MrgprA3 subset as an additional population of neuronal drivers likely acting via CGRP secretion. Cohen and colleagues demonstrated that optogenetic activation of TRPV1-expressing neurons initiated cutaneous IL-17-dependent inflammation via the neuropeptide CGRP^12^. CGRP has been proposed to initiate IL-17 responses by eliciting IL-23 from dermal CD301b+ cDC2s^13^. Moreover, nociceptor-derived CGRP was recently shown to promote *Il1b* expression in dendritic cells and their migration to inflamed tissues ^15^. Further, scRNA sequencing data indicates that MrgprA3+ neurons express *Calca* mRNA transcripts (encoding CGRP)^20^, but the biological role for this finding has remained unexplored. Our scRNAseq data revealed that multiple skin myeloid subsets express receptor components for CGRP, among other neuropeptides, indicating that myeloid cells are poised to directly respond to neuron-derived signals. TRPV1 is also expressed on non-neuronal cells including keratinocytes and immune cells, which regulates their function ^62, 63, 64^, but to date, MrgprA3 is not known to be expressed on non-neuronal cells. However, other Mrgpr family members, such as MrgprB2/X2 can be found in non-neuronal cells (Mast cells) and modulate itch and inflammatory responses^16, 25, 42, 43^, so such a possibility cannot be entirely ruled out for MrgprA3.

The close association of MrgprA3 neurons to skin APCs at baseline prompted us to examine the effects of activating MrgprA3-expressing neurons on skin architecture and immune status, which led to a novel connection between CGRP signaling and myeloid IL-33 expression. Critically, optogenetic or pharmacological activation of MrgprA3+ neurons selectively reduced IL-33 protein levels in tissue macrophages and cDC2s, while a complementary loss-of-function approach by DT injection of mice bearing DTR in MrgprA3 neurons^11^ did the opposite, suggesting that neuronal control of myeloid IL-33 occurs during homeostasis. Interestingly, CGRP inhibition prevented MrgprA3-induced neurogenic inflammation and restored IL-33 levels in myeloid cells, suggesting that CGRP release induced by MrgprA3 activation regulated myeloid derived IL-33. Similarly, CGRP was sufficient to suppress IL-33 protein levels in BMDMs and BMDCs. By contrast, CGRP has been shown to promote IL-33 expression in muscle cells^65^, similar to our results showing enhanced IL-33 expression in fibroblasts following MrgprA3 stimulation suggesting cell-specific dynamic regulation of IL-33 by neuronal afferents. When an *in vitro* approach was employed exposing BMDMs to supernatant from CQ-stimulated neurons, this resulted in their enhanced expression of inflammatory cytokines. Critically, RAMP1 deficiency only partially reduced the induction of pro-inflammatory cytokines induced by MrgprA3 neuro-derived soluble factors. RAMP1 and Calcr components of the CGRP receptor both make up the selective binding site for CGRP and RAMP1 is required to traffic the receptor complex to the plasma membrane^66^, so its loss alone should render cells unable to respond to CGRP. Alternatively, mediators like glutamate or HMGB1 are released by broadly defined populations of sensory neurons when activated and can modulate immune cell function^16, 17, 25, 67^. Thus, it is likely that mediators in addition to CGRP can enhance pro-inflammatory cytokine release by BMDMs observed following MrgprA3 neuron stimulation. Identification of other soluble mediators released by MrgprA3 neurons with immunoregulatory functions will require future studies.

One of the associated effects of manipulating MrgprA3 neurons was an alteration in myeloid expression of IL-33 which appears to shape the inflammatory potential of myeloid cells. After being released in the extracellular milieu, IL-33 exerts pleiotropic cellular effects after signaling through its receptor ST2 expressed in several lymphoid and myeloid cell lineages, including macrophages^68^. Indeed, macrophages were previously shown to respond to exogenous IL-33, which promotes pro-inflammatory cytokine expression and metabolic reprogramming^69^. Thus, we used two complimentary approaches to discern the effects of cell-intrinsic IL-33 from secreted IL-33 that signals via ST2. Blocking of ST2 or ST2 genetic deficiency did not exacerbate cytokine secretion by BMDMs. Further, treatment of IL-33-deficient BMDMs with rIL-33 did not restore their cytokine secretion to levels of control BMDMs. In contrast, IL-33 overexpression hindered the ability of macrophages to secrete pro-inflammatory cytokines. Consistent with these data, it was recently shown that IL-33 gene deletion altered differentiation of a subset of basal epithelial stem cells independently of its receptor ST2^70^, further suggesting that cell-intrinsic IL-33 can have a distinct biological role from secreted IL-33. Although different myeloid lineage cells (i.e. mast cells, macrophages, and dendritic cells) can produce IL-33 at low, yet biologically important levels, whether this IL-33 content could be dynamically regulated remained unknown. Moreover, whether the chromatin binding and histone localization of IL-33 serves any biological role other than sequestration is a long-standing unanswered question. IL-33 was first described as a nuclear-tethered cytokine constitutively found within the nuclei of cells that define tissue architecture and structural support including epithelia, endothelia, and fibroblast populations^26^. Consistent with this literature^46^, we find that skin fibroblasts presented with significant levels of IL-33 protein and transcript under basal conditions and that skin epithelial cells and endothelial cells have mostly IL-33 protein, but low levels of *Il33* transcripts. It has been long held that these cells only release IL-33 following tissue injury, but numerous reports have suggested that IL-33 can be found within the cytoplasm and released through pore forming proteins such as Perforin-2 in cDC, GSDMC in intestinal epithelial cells, and GSDMD in macrophages and hepatic cells^29, 71, 72, 73^. These mechanisms are remarkably similar to release of IL-1β through GSDMD pores, implying that IL-1 family cytokines use pore forming proteins for their release. Irrespective of how release is mediated, our data show that healthy skin contains multiple subsets of hematopoietic and non-hematopoietic lineage cells expressing IL-33 at different protein or transcript levels. Among myeloid subsets evaluated, only Langerhan cells, macrophages, mast cells, and cDC2 contained IL-33 protein and transcript during baseline conditions. This diversity in transcript vs protein content supports an emerging concept that biological roles served by IL-33 (i.e. promoting inflammation vs. immunoregulation) are highly dependent upon cellular context^29^. Indeed, data show that a subset of itch-specific neurons expressing MrgprA3 extended their afferents near skin-resident APCs that lacked IL-33, which inspired a hypothesis that activation of MrgprA3+ neurons could potentially modulate the cytokine profile of tissue resident myeloid phagocytes.

Investigation of whether myeloid-intrinsic IL-33 deficiency disrupted skin homeostasis, surprisingly revealed that mutant animals spontaneously developed epidermal thickening, excess keratinocyte turnover, and accumulation of cornified envelope proteins loricrin and desmoglein-1. These phenotypic alterations were associated with substantial alterations in different skin-resident lymphoid populations, most notably, increased IL-17+ γδ T cell responses, which were also increased in CX3CR1-IL-33KO mice. The alterations to skin homeostasis observed in the context of myeloid IL-33 regulation prompted us to investigate if cutaneous anti-helminth immunity could be altered in these settings. Remarkably, loss of myeloid-derived IL-33 prevented the skin entry of *S. mansoni* parasites and likely mitigated parasite migration upon tissue entry as shown by reduced pulmonary tissue larval load. Curiously, Langerin-mediated IL-33 ablation did not alter IL-17+ γδ T cell responses, further indicating cell-specific contributions of IL-33 expression to skin homeostasis. Recent work demonstrated that deficiency in ST2+ Treg populations was associated with increased γδ T cells in the lungs^48^. Further, our group previously showed that CD11c-IL-33KO mice have diminished numbers of GATA3+ and ST2+ Treg in the small intestine ^29^. Consistent with these data, CD11c-IL33KO mice had diminished GATA3+ Treg and ILC2 populations, suggesting that IL-33 production by myeloid cells restrains cutaneous type 17 cytokine responses while supporting GATA3+ skin lymphocytes. Nonetheless, lack of ST2-expressing Tregs was not sufficient to drive alterations in dermal γδ T cells or provide resistance against *S. mansoni* skin entry. A recent study showed that cutaneous IL-17 responses were exacerbated when type 2 cytokine signaling was abolished in cDC2^74^, suggesting that bidirectional regulatory mechanisms maintain the balance of type 2/type 17 cytokine skin immunity. γδ T cells are well-recognized to direct keratinocyte responses in the context of skin inflammatory conditions like psoriasis or during wound healing^3, 4, 5^. Furthermore, interactions between keratinocytes and subsets of γδ T cells were recently recognized to maintain epidermal integrity at the steady-state^75^. Accordingly, we found that neutralization of IL-17 and IL-23 reversed epidermal thickening, cutaneous cell proliferation, and resistance to *S. mansoni* observed in the absence of myeloid-IL-33, suggesting that myeloid cells influence keratinocyte homeostasis by limiting the magnitude of γδ T cell responses. Whether the IL-17/IL-23 axis also restricts larval tissue migration in the context of myeloid IL-33 deficiency will be the subject of further studies. Although type 1 immunity was previously shown to mediate early responses against *S. mansoni 76*, our data show an IFN-γ independent mechanism of innate resistance that restricts the initial entry of *S. mansoni* cercariae into the skin of CD11c-IL-33KO mice. Cercarial skin infection was previously shown to rapidly enhance IL-33 expression in the tissue and repeated cercarial exposures promote hyporesponsive immune responses^77, 78, 79^. While the cellular source of IL-33 was not evaluated in this case^77^, we speculate that *S. mansoni* parasites could potentially manipulate IL-33 expression in specific cellular compartments to suppress cutaneous immunity.

To investigate potential explanations for altered skin homeostasis in the absence myeloid-derived IL-33, we used scRNA-sequencing to solely focus on APC subsets. Loss of IL-33 in various APCs augmented the proportion of several myeloid cell clusters including macrophages, cDC1, cDC2, and immature/monocyte-like cDC. Transcriptionally, these clusters were enriched for expression of cytokines and chemokines known to regulate the activity of skin-resident lymphocytes such as IL-1β, TNFα, and Ccl2 ^3, 50, 51, 53^. Moreover, myeloid clusters expressed chemokines previously reported to promote myeloid cell recruitment including Ccl2 and Ccl9 ^54, 55^. Ablation of myeloid IL-33 increased pro-inflammatory cytokine and chemokine expression of at baseline including *Il1b, Tnf, Ccl2* and *Ccl9* in both cDC2 and macrophage clusters, suggesting that IL-33 may intrinsically regulate myeloid cell cytokine and chemokine expression patterns.

IL-33 binds to the tails of histones H2A and H2B which helps to maintain chromatin structure^56, 80^. Although, nuclear sequestration is a well-defined mechanism to restrict IL-33 mobilization and release^81^, a direct role for IL-33 in maintaining chromatin conformation that impacts gene transcription has remained elusive ^82^. We performed ATAC-seq analysis to evaluate whether loss of IL-33 differentially altered chromatin conformation in BMDMs vs. fibroblasts grown *in vitro* to remove the environmental stimuli experienced by skin-resident cells. Surprisingly, even in their quiescent state, IL-33-deficient macrophages presented with increased accessibility at multiple promoter regions compared to control cells. Pathway analysis showed that accessible regions in IL-33-deficient macrophages were related to Ras/kinases, proteasome/ubiquitination, metabolism/replication, immune surface/cytokine signaling and Inflammation gene sets. Macrophage-specific IL-33 deletion enhanced accessibility at *Tnf, Il23a,* and *Il1b* loci, but markedly reduced accessibility at the *Arg1* locus, the latter of which is a well-defined gene expressed by alternatively activated (M2) macrophages. Altogether, these data imply that IL-33 shapes chromatin conformation in macrophages and perhaps other myeloid lineage cells, which serve different biological roles as compared to fibroblasts. To evaluate whether these genomic alterations were evident at the post-translational level, we used *in vitro* approaches to show that IL-33 deficient BMDMs secreted higher levels of proinflammatory cytokines at baseline and when activated with LPS or ATP compared to their IL-33 sufficient counterparts. Similar results were observed when CD11c-specific IL-33KO BMDCs were exposed to LPS or ATP.

In sum, we propose that the alarmin cytokine IL-33 serves a cell-intrinsic role in shaping chromatin accessibility and transcriptional identity of cutaneous myeloid APC under basal conditions. Activation of itch-sensing MrgprA3 neurons re-shapes myeloid APC activity, at least in part, by regulating IL-33 expression, to rapidly unleash IL-17 dominant cutaneous inflammatory response upon pathogen encounter. Taken together, this study highlights a sensory neuron-myeloid APC circuit that takes advantage of how these distinctive cell types differ in response times, modes of sensing, and effector functions to promote barrier regulation and infection control in cutaneous microenvironments.

## MATERIALS AND METHODS

### Study design

The objectives of this study were to determine whether itch-sensing MrgprA3+ neurons regulate cutaneous inflammation by modulating myeloid cell cytokine expression and if IL-33 regulates myeloid cell functions to maintain tissue homeostasis. Experiments using mice aimed for group sizes of four to eight mice (matched for age and sex) and were repeated two to three times to assure reproducibility. Sample sizes were first defined by previous experience from this and other laboratories and then corroborated by Power analysis. Mice were tattooed for identification, with experimental groups randomized across cages to account for any microisolator effects. Parasite infection experiments and cell culture experiments were done using sex-matched male or female mice. To address subjectivity during the study, all animal cages and experimental samples/groups were assigned a letter number code to ensure that experiments were conducted in a blinded manner. Justification for removal of any outliers was solely based on Grubb’s test. For all flow cytometry experiments, we included negative controls, such as fluorescence minus one controls, to establish reliable and reproducible gates for each marker.

### Mice and experimental procedures

IL-33^fl/fl^ IRES-GFP mice were kindly donated by Paul Bryce’s laboratory and are currently available at Jackson Laboratories (B6(129S4)-Il33^tm1.1Bryc^/J). B6.Cg-Tg(Itgax-cre)1–1Reiz/J (CD11c Cre), B6.Cg-Gt(ROSA)^26Sortm32(CAG-COP4*H134R/EYFP)Hze^/J (Ai32), B6.Cg-Gt(ROSA)26Sor ^rtm9(CAG-tdTomato)Hze^/J (ROSA-TdT), B6J.B6N(Cg)-*Cx3cr1*^*tm1.1(cre)Jung*^/J (Cx3Cr1 Cre), C57BL/6-Gt(ROSA)26Sor^tm1(HBEGF)Awai/J^(ROSA26iDTR), and B6.C-Tg(CMV-cre)1Cgn/J (CMV Cre) mice were purchased from the Jackson Laboratories. Tg(Mrgpra3-GFP/cre)#Xzd (MrgprA3 Cre) mice were acquired from Wenqin Luo and Ishmail Abdus-Saboor laboratory. Tissues from Il1rl1^−/−^ mice were provided by Edward Behrens and Taku Kambayashi laboratory. Vijay Kuchroo’s lab and Kathleen Caron’s lab provided tissues to generate BMDMs and BMDCs from RAMP1KO mice. Foxp3^YFP-Cre^ mice were provided by Christopher Hunter. Dr. Botond Igyártó provided Langerin Cre mice. Conditional KO or optogenetic strains were bred to homozygosity and compared with age- and gender-matched controls from a separate set of breeders housed in the same facility. Co-housed Cre negative littermate controls were used for optogenetic and neuronal ablation experiments. All mice were housed under specific pathogen–free conditions at University of Pennsylvania. All procedures were approved by the Institutional Animal Care and Use Committee of the University of Pennsylvania (protocol 805911). For depletion of phagocytic cells, Standard Macrophage Depletion Kit (Clodrosome^®^ + Encapsome^®^, Encapsula NanoSciences, cat# CLD-8901) was used similar to previously described^83^. 20uL of vehicle- or clodronate-loaded liposomes (5mg/mL) were injected intradermally in the right or left ear of the same mouse to optimize each mouse as its own internal control. Liposome injections were performed 3 and 1 day prior to optogenetic stimulation as well as 1 day after the first photostimulation session. For cytokine neutralization experiments, mice were treated intraperitoneally (i.p.) with 500μg of neutralizing antibody against IL-17 (Bioxcell, cat# BE0173), IL-23 (Bioxcell, cat# BE0313), or IFNγ (Bioxcell, cat# BE0055) every 3 days for 10 days prior to cercarial exposure or tissue processing. Control mice were treated with the same amount of the appropriate isotype antibody. For CGRP inhibition, mice were treated with vehicle or 0.5μg of CGRP_8–37_ dissolved in 10μL of PBS 30mins prior to optogenetic stimulation. For chloroquine (CQ) intradermal injections, mice were anesthetized with isofluorane and 200μg of chloroquine (Sigma) dissolved in in 10μL of PBS were injected intradermally into the ear pinnae as indicated previously^32^. Mice were euthanized by CO2 for all tissue recovery procedures following AVMA guidelines.

### Optogenetic stimulation

Mice were anesthetized with isoflurane (1–4%) and maintained on a heating pad during photostimulation. Ear thickness was assessed using calipers, taking the average of 3–5 consecutive measurements as the value for each mouse. Optogenetic stimulation was performed using a 473nm 100mW diode-pumped solid state (DPSS) laser (SLOC Lasers, Shanghai, China), with attached power supply. An optical cannula aimed at the dorsal ear was placed 1–2 cm from the surface of the skin. Stimulation was performed with a sinus waveform generator to pulse 1Hz, pulse length for 30 minutes with a power density of 8–10 mW/mm^2^. Mice underwent five 30-minute stimulation daily sessions.

### Isolation of *Schistosoma mansoni* cercariae

*Biomphalaria glabrata* (NMRI strain) and *Schistosoma mansoni* (NMRI strain were provided by the NIAID Schistosomiasis Resource Center of the Biomedical Research Institute (Rockville, MD) through NIH-NIAID Contract HHSN272201700014I. NIH: Biomphalaria glabrata (NMRI) exposed to Schistosoma mansoni (NMRI). Maintenance and isolation of *S. mansoni* was performed as described in^36^. Previously exposed *Biomphalaria glabrata* (NMRI strain) snails were placed under light for 1–1.5hrs at 29°C. Then supernatants containing infectious *S. mansoni* cercariae were collected, filtered, and concentrated to a concentration of 100–150 cercariae in 0.1mL. For counting, live cercariae were immobilized by adding 50μL of iodine solution.

### Skin exposure to *Schistosoma mansoni* cercariae

Mice were anesthetized with Ketamine (70–100mg/kg)/Xylazine (5–12mg/kg) cocktail (i.p., 100μL/10g mouse). An infection ring was placed over the exposure area (ear pinnae) and surrounded by water-impermeable Vaseline. Then, a 100μL inoculum of 100–150 freshly isolated *S. mansoni* cercariae was placed over the skin for 20–30mins. Next, inoculum was resuspended, removed from the skin and the number of remaining cercariae present in the recovered inoculum was quantified.

### Quantification of lung *S. mansoni* parasites

For lung larvae (schistosomulae) quantification^36^, mice were infected with 500 *S. mansoni* cercariae by percutaneous infection in the ear. 6 days post-infection, lungs were recovered, mechanically digested in RPMI+10%FBS and incubated at 37°C for at least 3hours. Then cell suspensions were passed through a cheesecloth. Larval burden was determined by microscopic quantification.

### Skin digestion and preparation for flow cytometric analysis.

Ear skin explants were surgically dissected and processed similar to previously reported. Skin biopsies were mechanically dissociated and then incubated with skin digest solution containing 2mg/ml of collagenase XI (Sigma-Aldrich), 0.5mg/ml hyaluronidase (Sigma-Aldrich), and 0.1 mg/ml DNase (Roche) dissolved in DMEM-F12 containing 2.5% FBS for 25mins at 37°C with constant agitation. Skin homogenates were then passed through an 18-gauge needle 3–5 times and incubated at 37°C for additional 20mins. Then, cell preparations were passed again through an 18-gauge needle and filtered twice through 100μm and 40μm filters. Cells were then resuspended in RPMI supplemented with 10% FBS and incubated with Cell Activation Cocktail (with Brefeldin A) for 5–6hours. Then, skin exudates were stained for live/dead cell exclusion using LIVE/DEAD^™^ Fixable Aqua Dead Cell Stain Kit following manufacturer’s protocol. Fc Block was performed for 15mins at room temperature followed by surface marker staining for 25 mins on ice. Next, eBioscience^™^ Foxp3 / Transcription Factor Staining Buffer Set was used according to manufacturer’s protocol. Intracellular staining was done for 1–1.5hrs on ice. Cells were analyzed with a BD Symphony A3 Lite.

### Dorsal Root Ganglia (DRG) neuronal cultures and stimulation.

Lumbar and thoracic dorsal root ganglia (DRG) from 7–10-week-old mice were isolated as previously described^84^ and then enzymatically digested at 37°C for 20mins with 2.5 mg/mL of dispase II and 1.25 mg/mL of collagenase A dissolved in DMEM supplemented with 1% FBS. Cells were first dissociated with a 1mL pipette tip 20–30 times and then incubated at 37°C for additional 20mins. Next, DRG were triturated with a 23G and 27G needle 3–5 times and enzymes were neutralized with F12 media supplemented with 10% FBS, 2 mM glutamine and penicillin/streptomycin. Cell suspensions were layered in a 2-phase Percoll gradient (12% over 28%) and centrifuged at 1300g for 10mins. The fraction enriched with cell bodies was lysed of red blood cells and plated in 96-well plates previously coated with 10μg/mL laminin and 20μg/mL poly-lysine dissolved in HBSS without Ca^2+/^Mg^2+^. Cells were cultured in DMEM-F12 media containing 50 ng/mL NGF (R&D Systems) and 10μM cytosine arabinoside (Sigma-Aldrich), to restrict growth of non-neuronal cells. Cultures were maintained for 3 days, with media changed every 2 days. NGF was removed from the media prior to stimulation. On the day of the experiment, neuronal cultures were stimulated with 100μM Chloroquine (Sigma-Aldrich) for 1 hour. Cell-free supernatants were recovered and immediately used to stimulate BMDM cultures.

### Bone marrow derived macrophage (BMDM) assays.

Bone marrow (BM) cells from the femur and tibia were isolated and red blood cells were lysed. BM cells were cultured in DMEM-F12 media supplemented with 10%FBS and 20% supernatant derived from CMG14–12 (M-CSF producing) cell line for 5–7 days prior to experiments. BMDM polarization was assessed by cell morphology and later confirmed by cell surface expression of CD64, F4/80, CD11b, and CD11c. For some experiments, BMDMs were exposed to neuron-derived supernatants overnight. On the day of experiment, cells were exposed to vehicle, 50 or 250ng/mL of LPS (Sigma-Aldrich) for 6–8hours. BD GolgiPlug™ Protein Transport Inhibitor (containing Brefeldin A) and BD GolgiStop^™^ Protein Transport Inhibitor (containing Monensin) were added in the last 5 hours of stimulation. Cells and supernatant were recovered to be analyzed for cytokine expression by flow cytometry and cytokine concentration by ELISA, respectively. For IL-1β release, BMDMs were treated with 250ng/mL of LPS (Sigma-Aldrich) for 4 hours followed by exposure to vehicle, 0.5 or 2.5mM of ATP(Sigma-Aldrich). Supernatants were assessed for cytokine concentration by ELISA. For some experiments, control BMDMs were treated with 500ng/mL of neutralizing anti-ST2 antibody (R&D systems) or CD11c-IL-33KO BMDMs were exposed to 500ng/mL of mouse rIL-33 (R&D systems) 24hours prior to LPS stimulation.

### BMDM Transfection

WT or CMV-IL-33KO BMDMs were cultured for three days followed by enzymatic and mechanic detachment using 1X trypsin/EDTA solution (Thermofisher). As described in the literature^85^, BMDMs were transfected with 3μg of empty mCherry- or IL-33-mCherry plasmid generated by VectorBuilder. Transfections were performed using Amaxa^®^ Mouse Macrophage Nucleofector^®^ Kit and Nucleofector II/2b device (Lonza) following manufacturer’s procedures and cells were plated in DMEM-F12 media supplemented with 10%FBS and 20% supernatant derived from CMG14–12 (M-CSF producing) cell line. Two days post-transfection, mCherry plasmid and LPS-induced cytokine expression was determined by flow cytometry. IL-33 in the supernatant was also determined by ELISA (R&D systems).

### Primary fibroblasts culture.

Primary fibroblasts were generated as previously described^86^. Ear and tail skin were disinfected with 70% EtOH and mechanically dissociated with scissors and digested with 2mL of a 2.5mg/mL Collagenase D (Roche) and 1.25mg/mL of Pronase (Sigma) resuspended in 10%FBS-supplemented RPMI. Tissues were incubated for 90mins at 37C, followed by mechanical dissociation and filtration through a 70μm cell strainer. Cell suspensions were washed with RPMI+10%FBS+1XP/S+250μg/mL of amphotericin B and plated in 10mL of the same medium with media change every three days. Subcultures of fibroblasts were generated four days postseeding by enzymatically separating cells using 1X trypsin-EDTA mix, washed and replated in RPMI+10%FBS. Fibroblasts were used 10 days post-seeding.

### Immunofluorescence

Abdominal tissue samples were fixed in cold 4% PFA for 2 hr at room temperature, washed with 1XPBS for 1–4 hrs. and left in PBS containing 30% sucrose overnight. Samples were then embedded with OCT and 6-μm-thick sections were cryosectioned. Tissue sections were first incubated for 90 minutes at room temperature in blocking buffer (1XTBS containing 5% normal donkey serum, Fc Block, 1%BSA, and 0.3% Triton X-100). After rinsing three times with 1XTBS, sections were incubated in primary antibodies diluted in blocking buffer at 4°C overnight. Sections were rinsed and incubated with secondary antibodies diluted in blocking buffer for 120 min. at room temperature. Sections were incubated with DAPI diluted in 1XTBS 1%BSA for 10–15 min. at room temperature. Sections were then mounted on glass slides with Fluoroshield mounting medium. The primary antibodies used for immunofluorescence staining are chicken anti-mouse Keratin 5 Polyclonal Antibody (1:1000; BioLegend); rat anti-mouse MHC Class II antibody [M5/114] (1/100; Abcam); rabbit anti-mouse Loricrin antibody (1:400; BioLegend); rabbit anti-mouse Desmoglein 1/DSG1 antibody [EPR6766(B)] (1:200; Abcam); rabbit anti-mouse Ki67 antibody (1:200; Abcam); biotinylated mouse anti-mouse Tubulin β 3 (TUBB3) Antibody (1:200; BioLegend); DAPI Solution (1:1000; ThermoFisher). The secondary antibodies used are Alexa Fluor 594 Donkey anti-chicken IgY, Alexa Fluor 488 Donkey anti-chicken, Cy3 Donkey anti-rat IgG, Cy3 Donkey anti-rabbit, and Cy3 Streptavidin, all at 1:500 dilution. Detection of fluorescence was observed under DAPI, GFP, Cy3 filters on a Leica DM6000 motorized upright microscope and DAPI, GFP, Cy3, Cy5 and Texas Red filters on a Leica DMI 6000 inverted microscope (Leica TCS SP8 WLL Confocal). Exposure times and fluorescence intensities were normalized to appropriate control images. We photographed fluorescence channels separately, merged them together, and overlaid them atop the corresponding images. Distance of MHC-II+ cells to the closest A3+ neuronal fiber was determined using the ‘scale bar’ function of the Leica Application Suite X Version 5.1.0.25446 drawing a straight line from the cell cytoplasm to the nearest A3+ nerve fiber.

### ELISA

Cell-free supernatants were used to measure cytokine concentration levels using the commercially available assays listed in Key Resources Table. Skin biopsies were homogenized in RIPA buffer containing 1x Proteinase cocktail inhibitor (ThermoFisher). Protein concentration was determined using the Pierce BCA Protein Assay Kit (ThermoFisher). Then 1–2mg of protein were used to determine cytokine levels using the assays stated above.

### Quantitative real-time PCR

RNA from tissues were isolated using NucleoSpin RNA Plus kit (Macherey-Negel, Dueren, Germany), and cDNA was generated with Maxima H Minus Reverse Transcriptase (Thermo Fisher). Reverse transcription PCR data were analyzed using the 2^ΔΔct^ method with the SYBR Green Chemistry (ThermoFisher) reagent, with *Gapdh* gene serving as the endogenous housekeeping gene. Samples were normalized to naive controls. Quantitative real-time PCR was run and analyzed on CFX96 platform (Bio-Rad, Hercules, CA). Primers used are listed in Key Resources Table.

### Skin RNA extraction and preparation for bulk RNAseq

Skin biopsies were homogenized and tissue RNA was isolated using the Qiagen RNAeasy plus kit (Qiagen). RNA integrity was measured with an Agilent RNA TapeStation, and concentration measured with the qubit fluorometer (Invitrogen). Samples with RIN values greater than 7 were used for sequencing, and RNA was normalized to 500ng per sample. Libraries were generated using the Illumina stranded mRNA prep kit (Illumina) and sequenced using an Illumina NextSeq 550 in a single-end 75-base pair (bp) read configuration, at a depth of at least 30 million reads per sample. The output reads were trimmed to remove adapter content using trimmomatic^87^. Trimmed reads were aligned to the mouse GRCm38 reference genome using STAR v2.6.1c, followed by gene count generation using htseq-count^88, 89^. Read counts were normalized and compared for differential gene expression using DESeq2 v1.22.1 with significance at a false discovery rate-adjusted P < 0.05. To identify enriched functional pathways, GSEA software was used with the pre-ranked gene list setting determined by log2 fold-change values, compared to the Molecular Signatures Database (MSigDB) hallmark gene sets database^90^.

### Single-cell RNA sequencing

Abdominal skin-resident myeloid cells from control or CD11-IL-33KO mice were sort-purified as Live CD45+ MHC-II+ CD11c+ cells using an BD FACS Aria II sorter. Sort-purified cells were resuspended in PBS supplemented with 10%FBS to achieve a target cell concentration of 700 to 1200 cells per μl. Cell viability was determined by trypan blue exclusion and only samples with >85% viability were processed. Next-generation sequencing libraries were prepared using the 10x Genomics Chromium Single Cell 3’ Reagent kit v3 (10X Genomics) per manufacturer’s instructions. Libraries were uniquely indexed using the Chromium dual Index Kit, pooled, and sequenced on an Illumina NovaSeq 6000 sequencer in a paired-end, dual indexing run. Sequencing for each library targeted 20,000 mean reads per cell. Data was then processed using the Cell Ranger pipeline (10x Genomics, v.6.1.2) for demultiplexing and alignment of sequencing reads to the mm10 transcriptome and creation of feature-barcode matrices.

Data were further processed with Seurat 4.0 R package^91^. For quality control, only genes expressed in at least 3 cells and cells expressing at least 200 genes were included. Cells expressing >10% mitochondrial genes were excluded from the downstream analysis. Data were normalized and scale using default parameters and the number of principal components were estimated using *RunPCA* followed by *ElbowPlot*. Uniform Manifold Approximation and Projection (UMAP) was used for dimensionality reduction and performed using *RunUMAP*. Markers for cell clusters were identified using the *FindAllMarkers* function equipped in the Seurat and cell types were annotated manually using canonical markers. To further resolve cell subtypes (e.g., B cells), the subset function in Seurat was used, with subset of cells subjected to a second round of principal components identification and dimensional reduction as the same as above. We deployed the processed results in Shinny App for further exploration.

### ATAC-seq

Control or CD11c-IL-33KO BMDMs were differentiated as mentioned above. Then, libraries from cells were prepared using the Illumina Tagment DNA Enzyme and Buffer Small Kit (Ilumina) according to manufacturer’s protocol. Sequencing was performed using a third-party company (Novogene). For the data analysis, two of public samples (Accession: GSM4564315 and GSM4564316)^92^ were added into the control group. The sequence adopters of the FASTQ files were trimmed using Trimmomatic version 0.39 program^87^ and then align to mouse reference genome (mm10) using R subread version 2.10.1 R package^93^. The mitochondrial reads and PCR duplicates were removed using SAMtools version 1.13 for the downstream analysis^94^. The reads with high alignments (MAPQ score >30) were kept (the public data with MAPQ score >10) ^92^. All reads aligning to the positive strand were offset by +4 bp, and all reads aligning to the negative strand were offset −5 bp to adjust the Tn5 transposase binding site as a dimer and insert two adapters separated by 9 bp^95^. Peak calling were performed using MACS2 v.2.7.4^96, 97^ with piling up of paired--end fragment mode (--format BAMPE). and the peak files (bed) were filtered by removing the ENCODE black listed regions (https://www.encodeproject.org/files/ENCFF543DDX). Non-redundant peaks were identified and differential accessibility (DA) was determined using DESeq2 R package and the pathway analysis of the DA list was conducted using cinaR^98, 99^.

### Statistics

Results are shown as the mean ± s.e.m. P < 0.05 was considered as significantly different. Grubb’s test was used to identify outliers. Because these data were normally distributed, we used parametric statistical tests. Statistical analysis was performed using Student’s t-test for two groups, one-way ANOVA for three groups, or two-way ANOVA (ear thickness measurements), with appropriate post-hoc tests in Prism 10 (GraphPad Software).

## Extended Data

## Figures and Tables

**RESOURCES TABLE:** 

REAGENT OR RESOURCE	SOURCE	IDENTIFIER
Antibodies		
BUV395^™^ Rat Anti-Mouse Ly-6A/E Antibody	BD Biosciences	Cat# 563990. RRID:AB_2738527
BUV563^™^ Rat Anti-Mouse CD45 Antibody	BD Biosciences	Cat# 752412. RRID:AB_2873124
BUV737^™^ Rat Anti-Mouse CD11b Antibody	BD Biosciences	Cat# 612801. RRID:AB_2738811
Brilliant Violet 421^™^ anti-Mouse CD140a Antibody	Biolegend	Cat# 135923. RRID: AB_2814036
Brilliant Violet 605^™^ anti-Mouse CD11c Antibody	Biolegend	Cat# 117334. RRID: AB_2562415
Brilliant Violet 650^™^ anti-Mouse XCR1 Antibody	Biolegend	Cat# 148220. RRID: AB_2566410
Brilliant Violet 711^™^ anti-Mouse Ly6G Antibody	Biolegend	Cat# 127643. RRID: AB_2565971
Brilliant Violet 785^™^ anti-Mouse Ly6C Antibody	Biolegend	Cat# 128041. RRID: AB_2565852
GFP Antibody Fluorescein Conjugated	Rockland	Cat# 600-402-215
PerCP/Cy5.5 anti-Mouse CD31 Antibody	Biolegend	Cat# 102522. RRID: AB_2566761
Mouse IL-33 Alexa Fluor^®^ 647-conjugated Antibody	R&D systems	Cat# IC3626R
Alexa Fluor^®^ 700 anti-mouse I-A/I-E Antibody	Biolegend	Cat# 107622. RRID: AB_493727
APC/Cy7 anti-Mouse CD326 (Ep-CAM) Antibody	Biolegend	Cat# 118218. RRID: AB_2098648
FceR1 alpha Monoclonal Antibody (MAR-1), PE	ThermoFisher	Cat#12-5898-82.RRID:AB_466028
PE/Dazzle^™^594 anti-mouse CD172a (SIRPα) Antibody	Biolegend	Cat# 144016. RRID: AB_2565280
PE/Cyanine5 anti-mouse F4/80 Antibody	Biolegend	Cat# 123112. RRID: AB_893482
PE/Cyanine7 anti-mouse CD64 (FcγRI) Antibody	Biolegend	Cat# 139314. RRID: AB_2563904
BUV395^™^ Rat Anti-Mouse IL-17 Antibody	BD Biosciences	Cat# 565246. RRID:AB_2722575
BUV737^™^ Rat Anti-Mouse CD4 Antibody	BD Biosciences	Cat# 612844. RRID:AB_2738811
Brilliant Violet 421^™^ anti-mouse IL-33Rα (IL1RL1, ST2) Antibody	Biolegend	Cat# 145309. RRID: AB_2565634
Brilliant Violet 605^™^anti-mouse TCR γ/δ Antibody	Biolegend	Cat# 118129. RRID: AB_2563356
Brilliant Violet 711^™^ anti-Mouse CD90.2 Antibody	Biolegend	Cat# 105349. RRID: AB_2800564
Brilliant Violet 785^™^ anti-mouse IL-33Rα (IL1RL1, ST2) Antibody	Biolegend	Cat# 145321. RRID: AB_2860702
Gata-3 Monoclonal Antibody (TWAJ), Alexa Fluor^™^ 488, eBioscience^™^	ThermoFisher	Cat# 53-9966-42. RRID: AB_2574493
PerCP/Cy5.5 anti-Mouse CD19 Antibody	Biolegend	Cat# 152406. RRID: AB_2629815
APC anti-mouse IFN-γ Antibody	Biolegend	Cat# 505810. RRID: AB_315404
Ki-67 Monoclonal Antibody (SolA15), APC-eFluor^™^ 780, eBioscience^™^	ThermoFisher	Cat# 47-5698-82. RRID: AB_2688065
FOXP3 Monoclonal Antibody (FJK-16s), PE, eBioscience^™^	ThermoFisher	Cat#12-5773-82.RRID:AB_465936
PE/Dazzle^™^ 594 anti-mouse CD8a Antibody	Biolegend	Cat# 100762. RRID: AB_22564027
PE/Cyanine5 anti-mouse TCR β chain Antibody	Biolegend	Cat# 109210. RRID: AB_313433
IL-13 Monoclonal Antibody (eBio13A), PE-Cyanine7, eBioscience^™^	ThermoFisher	Cat# 25-7133-82. RRID: AB_2573530
IL-1 beta (Pro-form) Monoclonal Antibody (NJTEN3), eFluor^™^ 450, eBioscience^™^	ThermoFisher	Cat# 48-7114-82. RRID: AB_2574109
IL-6 Monoclonal Antibody (MP5-20F3), PerCP-eFluor^™^ 710, eBioscience^™^	ThermoFisher	Cat# 505204. RRID: AB_2573829
PE anti-mouse IL-12/IL-23 p40 Antibody	Biolegend	Cat# 109210. RRID: AB_315368
Keratin 5 Polyclonal Chicken Antibody, Purified	Biolegend	Cat# 905901. RRID: AB_2565054
Anti-MHC Class II antibody [M5/114]	Abcam	Cat# ab139365
Purified anti-Loricrin Antibody	Biolegend	Cat# 905104. RRID: AB_2616895
Anti-Desmoglein 1/DSG1 antibody [EPR6766(B)]	Abcam	Cat# ab124798
Anti-Ki67 antibody	Abcam	Cat# ab15580
DAPI Solution	ThermoFisher	Cat# 62248
Alexa Fluor 594 AffiniPure F(a’)_2_ Fragment Donkey Anti-Chicken IgY (IgG) (H+L)	Jackson ImmunoResearch Laboratories Inc.	Cat# 703-586-155
Cy3 AffiniPure F(ab’)_2_ Fragment Donkey Anti-Rat IgG (H+L)	Jackson ImmunoResearch Laboratories Inc.	Cat# 712-166-150
Cy3 AffiniPure F(ab’)_2_ Fragment Donkey Anti-Rabbit IgG (H+L)	Jackson ImmunoResearch Laboratories Inc.	Cat# 711-166-152
Biotin anti-Tubulin β 3 (TUBB3) Antibody	BioLegend	Cat#: 801212
Cy3 Streptavidin	Jackson ImmunoResearch Laboratories Inc.	Cat# 016-160-084
Alexa Fluor 488 AffiniPure F(ab’)_2_ Fragment Donkey Anti-Chicken IgY (IgG) (H+L)	Jackson ImmunoResearch Laboratories Inc.	Cat# 703-545-155
Chemicals, peptides, and recombinant proteins		
InVivoMAb anti-mouse IL-17A (Clone: 17F3)	Bioxcell	Cat# BE0173
InVivoMAb mouse IgG1 isotype control, unknown specificity	Bioxcell	Cat# BE0083
InVivoMAb anti-mouse IL-23 (p19) (Clone: G23-8)	Bioxcell	Cat# BE0313
InVivoMAb rat IgG1 isotype control, anti-horseradish peroxidase	Bioxcell	Cat# BE0088
InVivoMAb anti-mouse IFNγ (Clone: XMG1.2)	Bioxcell	Cat# BE0055
Mouse ST2/IL-33R Antibody	R&D Systems	Cat# AF1004
Recombinant Mouse IL-33 Protein	R&D Systems	Cat# 3626-ML-010
CGRP	Tocris	Cat# 1161
CGRP_8-37_	Tocris	Cat# 1169
Standard Macrophage Depletion Kit (Clodrosome^®^ + Encapsome^®^)	Encapsula NanoSciences	Cat# CLD-8901
Diphtheria Toxin (unnicked) from Corynebacterium diphtheriae	Cayman Chemical	Cat# 19657
Collagenase from *Clostridium histolyticum*	Sigma-Aldrich	Cat# C7657
Hyaluronidase from bovine testes	Sigma-Aldrich	Cat# H3506
DNase I from bovine pancreas	Sigma-Aldrich	Cat# 11284932001
Cell Activation Cocktail (with Brefeldin A)	Biolegend	Cat# 423304
Collagenase A from *Clostridium histolyticum*	Sigma-Aldrich	Cat# 10103578001
Collagenase D	Roche	Cat# 11088866001
Pronase from *Streptomyces griseus*	Sigma-Aldrich	Cat# 10165921001
Dispase II	Sigma-Aldrich	Cat# D4693
Laminin mouse protein	ThermoFisher	Cat# 23017015
Poly-D-Lysine	ThermoFisher	Cat# A3890401
BD GolgiPlug^™^ Protein Transport Inhibitor (containing Brefeldin A)	BD Biosciences	Cat# 555029
BD GolgiStop^™^ Protein Transport Inhibitor (containing Monensin)	BD Biosciences	Cat# 554724
Chloroquine diphosphate salt	Sigma-Aldrich	Cat# C6628
eBioscience^™^ Foxp3 / Transcription Factor Staining Buffer Set	ThermoFisher	Cat# 00-5523-00
DMEM/F-12, powder	ThermoFisher	Cat# 12500062
RPMI 1640 Medium, powder	ThermoFisher	Cat# 31800-022
Percoll	Sigma-Aldrich	Cat# P1644
Trypsin-EDTA (0.05%), phenol red	ThermoFisher	Cat# 25300054
Maxima H Minus Reverse Transcriptase	ThermoFisher	Cat# EP0753
Recombinant Mouse beta-NGF Protein	R&D systems	Cat# 1156-NG-100/CF
Cytosine β-D-arabinofuranoside hydrochloride	Sigma-Aldrich	Cat# C6645
Lipopolysaccharides from Escherichia coli O111:B4	Sigma-Aldrich	Cat# L4391
Adenosine 5′-triphosphate disodium salt hydrate	Sigma-Aldrich	Cat# A7699
SsoAdvanced Universal SYBR^®^ Green Supermix	Biorad	Cat# 172-5274
RIPA Lysis Buffer, 10X	Sigma-Aldrich	Cat# 20-188
Halt^™^ Protease Inhibitor Cocktail (100X)	ThermoFisher	Cat# 78430
16% Paraformaldehyde (formaldehyde) aqueous solution	Electron Microscopy Sciences	Cat#15710-S
Normal donkey serum	ThermoFisher	Cat# NC9624464
Fluoroshield Histology Mounting Medium	Sigma-Aldrich	Cat# F6182
Ketaset (Ketamine hydrochloride injection)	Zoetis	NADA# 043-304
Anased (Xylazine injection)	AKORN	NADA# 139-236
Commercial Assays		
IL-17A (homodimer) Mouse Uncoated ELISA Kit	ThermoFisher	Cat# 88-7371-88
IL-23 Mouse Uncoated ELISA Kit	ThermoFisher	Cat# 88-7230-88
IL-1 beta Mouse Uncoated ELISA Kit	ThermoFisher	Cat# 88-7013-88
IL-22 Mouse Uncoated ELISA Kit	ThermoFisher	Cat# 88-7422-88
Mouse IL-33 DuoSet ELISA	R&D systems	Cat# DY3626
TNF alpha Mouse Uncoated ELISA Kit	ThermoFisher	Cat# 88-7324-88
CGRP (rat) EIA Kit	Cayman Chemicals	Cat# 589001
LIVE/DEAD^™^ Fixable Aqua Dead Cell Stain Kit, for 405 nm excitation	ThermoFisher	Cat# L34966
NucleoSpin RNA Plus kit	Macherey-Negel	Cat# 740984.25
RNeasy Plus Mini Kit	Qiagen	Cat# 74134
Illumina Tagment DNA Enzyme and Buffer Small Kit	Ilumina	Cat# 20034197
Ilumina stranded mRNA kit	Ilumina	Cat# 20040532
Pierce BCA Protein Assay kit	ThermoFisher	Cat# 23225
Chromium Single Cell 3’ Reagent kit v3	10X Genomics	Cat# PN-1000075
Mouse Macrophage Nucleofector^™^ Kit	Lonza	Cat# VPA-1009
Deposited Data		
Skin Bulk RNA-seq	This paper	GSE218826
CD11c+ MHC-II+ scRNA-seq	This paper	GSE218825
ATAC-seq	This paper	GSE218833
Experimental models: Organisms/strains		
Mouse: B6(129S4)-M33^tm1.1Bryc^/J (IL33fl/fl-eGFP)	The Jackson Laboratory	Strain #030619
Mouse: B6.Cg-Tg(Itgax-cre)1-1Reiz/J (Cd11c-Cre)	The Jackson Laboratory	Strain #008068
Mouse: B6.Cg-Gt(ROSA)^26Sortm32(CAG-COP4*H134R/EYFP)Hze^/J (Ai32)	The Jackson Laboratory	Strain # 024109
Mouse: C57BL/6-Gt(ROSA)26Sor^tm1(HBEGF)Awai/J^ (ROSA26iDTR)	The Jackson Laboratory	Strain #007900
Mouse: Tg(Mrgpra3-GFP/cre)#Xzd (MrgprA3^Cre^)	^11^	N/A
Mouse: Il1rl1^−/−^ mice (ST2KO mice)	^100^	N/A
Mouse: B6.Cg-Gt(ROSA)26Sor^tm9(CAG-tdTomato)Hze^/J	The Jackson Laboratory	Strain # 007909
Mouse: Ramp1^−/−^ mice	^101^	N/A
Mouse: B6J.B6N(Cg)-*Cx3cr1^tm1.1(cre)Jung^*/J	The Jackson Laboratory	Strain # 025524
Mouse: Langerin-Cre	^49^	N/A
Mouse: Il1rl1^tm1a(KOMP)Wtsi^	^102^	N/A
Mouse: Foxp3^YFP-Cre^	^103^	N/A
Mouse: B6.C-Tg(CMV-cre)1Cgn/J	The Jackson Laboratory	Strain # 006054
Parasite: *Schistosoma mansoni* (NMRI strain)	NIAID Schistosomiasis Resource Center	N/A
Snails: *Biomphalaria glabrata (NMRI)*	NIAID Schistosomiasis Resource Center	N/A
Oligonucleotides		
qPCR primer: *Il17a*-forward: 5’-ACTACCTCAACCGTTCCACG-3’	Primer Bank	PrimerBank: NM_010552
qPCR primer: *Il17a*-reverse: 5’-CACACCCACCAGCATCTTCT-3’	Primer Bank	PrimerBank: NM_010552
qPCR primer: *Il22*-forward: 5’-TGCTTCTCATTGCCCTGTG-3’	Primer Bank	PrimerBank: NM_054079
qPCR primer: *Il22*-reverse: 5’- TGGATGTTCTGGTCGTCACC-3’	Primer Bank	PrimerBank: NM_054079
qPCR primer: *Il23a*-forward: 5’-TGTGAAGATGGTTGTGACC-3’	Primer Bank	PrimerBank: NM_031252
qPCR primer: *Il23a*-reverse: 5’-GGCTATCAGGGAGTAGAGCA-3’	Primer Bank	PrimerBank: NM_031252
qPCR primer: *Il1b*-forward: 5’-GCAACTGTTCCTGAACTCAAC-3’	Primer Bank	PrimerBank: NM_008361
qPCR primer: *Il1b*-reverse: 5’-ATCTTTTGGGGTCCGTCAACT-3’	Primer Bank	PrimerBank: NM_008361
qPCR primer: *Gapdh*-forward: 5’-AGGTCGGTGTGAACGGATTTG-3’	Primer Bank	PrimerBank: NM_008084
qPCR primer: *Gapdh*-reverse: 5’-TGTAGACCATGTAGTTGAGGT-3’	Primer Bank	PrimerBank: NM_008084
pRP[Exp]-mCherry-CMV+intron>mIl33[NM_133775.3]	VectorBuilder	Cat# Ecoli (VB230327-1292tgd)
mCherry control vector pRP[Exp]-mCherry/Puro-CAG>ORF_stuffer	VectorBuilder	Cat# Ecoli (VB010000-9290tfx)-P
Instruments and equipment		
Symphony A3 Lite	BD Biosciences	N/A
Aria II	BD Biosciences	N/A
MITUTOYO Dial Thickness Gauge	Grainger	Part # 6NRC7
473nm DPSS Laser Nd:YAG	SLOC lasers	Model BL473T8-100FC
Power source	SLOC lasers	Model ADR-800A
1×2 MM Fiber Optic Coupler	Thor Labs	Part# TM200FS1B
Power and energy meter	Thor Labs	Part# PM100D
10 MHz Dual Channel Function/Arbitrary Waveform Generator	BX Precision	Part# 4053B
CFX96 system	Bio-Rad	Part# 3600037
Nucleofector^™^ II/2b Device	Lonza	Cat. #LO AAB-1001
Leica DM6000	Leica	N/A
Leica TCS SP8 WLL Confocal	Leica	N/A
Illumina NovaSeq 6000	Ilumina	N/A
Illumina NextSeq 550	Ilumina	N/A
Software and algorithms		
Flowjo 10.8.1	Flowjo	N/A
Prism 10	Graph Pad	N/A
Leica Application Suite X Version 5.1.0.25446	Leica	N/A
Cell Ranger pipeline v.6.1.2	10x Genomics,	N/A
Trimmomatic	GPL V3	N/A
STAR v2.6.1c	GPL V3	N/A
DESeq2 v1.22.1	LGPL	N/A
Gene Set Enrichment Analysis (GSEA)	UC San Diego	N/A
*Seurat* 4.0 R package	^91^	N/A
SAMtools version 1.13	^94^	N/A
MACS2 v.2.7.1	^97^	N/A
DESeq2 R package	^98^	N/A
cinaR	^99^	N/A

## Data Availability

All reagents generated or used in this study are available on request from the lead contact with a completed Materials Transfer Agreement. Information on reagents used in this study is available in the key resources table. All the data supporting the findings of the article are available within the main text or supplemental information. The published article includes datasets generated during this study. Original bulk RNA-seq, single cell RNA-seq, and ATAC-seq data has been deposited in GEO: GSE218834.
